# Social vulnerability among Brazilian children in early childhood: a scoping review

**DOI:** 10.1016/j.jped.2024.06.012

**Published:** 2024-08-17

**Authors:** Alcina M. Brito, Deisiane O. Souto, Luana C. Silva, Hércules R. Leite, Rosane L.S. Morais

**Affiliations:** a Universidade Federal dos Vales do Jequitinhonha e Mucuri, Programa de Pós-Graduação em Saúde, Sociedade e Meio Ambiente, Diamantina, MG, Brazil; b Universidade Federal de Minas Gerais (UFMG), Escola de Educação Física, Fisioterapia e Terapia Ocupacional (EEFFTO), Departamento de Fisioterapia, Programa de Pós-Graduação em Ciências da Reabilitação (PPGCR), Belo Horizonte, MG, Brazil

**Keywords:** Brazilian children, Early childhood, Social protection

## Abstract

**Objective:**

To identify, map, and describe studies involving Brazilian children in early childhood in situations of social vulnerability.

**Source of data:**

A scoping review including full articles published in Portuguese and English up to March 2023, with no temporal restrictions. Searches were conducted in the MEDLINE/PubMed, Scielo, EMBASE, Cochrane, Scopus, CINAHL, Web of Science, PEDro, and LILACS databases. Journal metrics, sample characteristics, study area, characterization of the situation of social vulnerability, and study outcomes were extracted.

**Summary of the findings:**

Seventy-six articles involving a total of 107.740 children in early childhood were included in this study. These studies presented relevant findings, including the temporal publication trend, the variability of social vulnerability indicators, the scarcity of intervention studies, and the fact that 100% of eligible studies were from the health area. Negative outcomes were associated with the condition of social vulnerability in almost the entire sample, reinforcing the need for government policies capable of protecting early childhood from the effects of social vulnerability.

**Conclusion:**

This scoping review mapped important findings involving Brazilian children in social vulnerability. It also identified literature gaps such as the need for intervention and multisectoral studies among health, education, and social protection.

## Introduction

Early childhood, from birth to six years of age, has been the subject of study and the focus of attention in various institutions in Brazil and worldwide in recent years. The increased interest in conducting studies directed at the first six years of life is motivated by the fact that this is a sensitive period of brain development, considered a window of opportunities for the individual's full development.^1^ This period requires special attention because there is greater potential for the development of cognitive, affective, and socio-emotional skills, considered as the foundation for later stages of development.^2^

The Brazilian population in the 0–6 age group is estimated at 19 million, representing 8.91% of the country's 211 million inhabitants.^3^ Among these, there is a portion living in poverty and extreme poverty. The Institute of Applied Economic Research (IPEA) pointed out another alarming data, that there was an increase of over one million people living in poverty in Brazil as a consequence of the COVID-19 pandemic.^4^ In addition to this statistic, a warning about the situation of childhood in the country is the statement by experts that children are disproportionately represented in the population indices affected by poverty. That is, the number of children is much higher than that of adults in situations of social vulnerability due to the context of poverty or extreme poverty to which they are exposed.^5^

The concept of social vulnerability presents variations depending on the study area in Brazil, demonstrating that there is no single or consensual definition of this term in the country.^6^, ^7^, ^8^ Vulnerability is characterized by a set of individual or collective factors that make individuals or communities more susceptible to illnesses or harms, associated with a lack of resources capable of providing protection.^9^ The United Nations International Children's Emergency Fund (UNICEF), in turn, uses the term multidimensional poverty to express a situation that goes beyond the lack of financial resources but is related to deprivations, exclusions, and vulnerabilities to which children are exposed.^9^ Eight indicators are evaluated in this poverty definition: food, education, income, sanitation, water, protection against child labor, housing, and information.^9^

For this study, the authors adopted the understanding of the National Social Protection Policy (PNAS), whose conception takes into account various factors beyond poverty or lack of financial resources.^10^ It defines that basic social protection should serve the population that is socially vulnerable due to factors such as poverty, lack of income, lack of access, or precarious access to public services. People with weakened social and affective bonds or who are subjected to discrimination based on gender, race, physical, mental, or intellectual disabilities, among other adverse situations.^10^

Children in social vulnerability are more exposed to adverse experiences, such as food insecurity, neglect in care, malnutrition, and violence. Such experiences tend to harm child development in all its domains: cognitive/language, affective-social, and motor.^11^ Anemia in infants due to iron deficiency poses a risk to short- and long-term development even when they undergo iron replacement therapy.^11^^,^^12^ There is evidence that malnutrition continues to be a serious public health problem that affects developing countries, being responsible, even in this century, for alarming mortality rates of children under five years of age around the world.^13^^,^^14^ Millions of children in early childhood living in developing countries are unable to reach their development potential due to a lack of access to health, nutrition, safety, protection, responsive care, and appropriate learning materials.^15^ Comprehensive studies involving underdeveloped and developing countries on different continents have been carried out with the aim of demonstrating how necessary care is in this age group, combating the effects of poverty and other risk factors on child development.^7^^,^^12^^,^^13^

The 1988 Federal Constitution grants Brazilian children the status of rights holders, as determined in its article 277: "It is the duty of the family, society, and the State to ensure, with absolute priority, the rights of children and adolescents to life, health, [...], as well as to protect them from all forms of negligence, discrimination, exploitation, violence, cruelty, and oppression".^16^ Since then, important steps have been taken to guarantee the rights of children, such as the Statute of the Child and Adolescent; the ratification, by Brazil, in September 1990, of the Convention on the Rights of the Child; and the approval, in 2010, of the National Plan for Early Childhood, the Legal Framework for Early Childhood, among others.^17^^,^^18^

It is known that an intersectoral effort involving health, education, and social protection is necessary to reach this audience effectively and efficiently.^19^^,^^20^ It can be observed that in Brazil, a legal framework has been produced to guarantee the protection of its children, as well as the implementation, over the past 30 years, of public policies aimed at consolidating this protection. However, there are questions about the practical results of these efforts: Do the studies produced in this area reveal what the country has achieved or done in terms of health, well-being, and the development of early childhood in situations of social vulnerability? What has been studied and published based on what is being done for the health, well-being, and development of early childhood in social vulnerability in Brazil? What are the outcomes of studies involving Brazilian children in early childhood in social vulnerability?

The authors consider it of fundamental importance to highlight what is documented in the literature regarding Brazilian children in early childhood in situations of social vulnerability. Therefore, the authors chose the literature review method by Scoping Review for this study. This type of research is suitable for answering broad questions and mapping the literature in a specific area of knowledge.^21^

## Methods

A Scoping Review was conducted following the methodological recommendations of the Joanna Briggs Institute (JBI) and the items of the Preferred Reporting Items for Systematic Reviews and Meta-Analyses (PRISMA) checklist- extension for scoping reviews.^22^ This method has well-defined criteria, and its main indication is to map scientific production by area of knowledge. It is capable of answering broad questions, identifying gaps in certain fields, synthesizing results, and making recommendations for new studies. The protocol for this Scoping Review prepared as provided in the JBI and PRISMA checklist, is registered on the Open Science Framework (OSF) platform DOI: 10.17605/OSF.IO/CU69Q.

Considering that this study involves Brazilian children, the authors chose to use the concept of early childhood defined and utilized in Brazil, which is the period characterized by the age range from zero to six years.^1^^,^^3^ Regarding the definition of social vulnerability, the authors used the one provided by the PNAS.^10^

### Search strategy

To structure the search strategy, the main question was adapted to the PCC mnemonic (P: Population; C: Concept; C: Context); corresponding respectively to Brazilian children, early childhood, and social vulnerability. Searches for eligible studies were conducted in the MEDLINE/PubMed, Scielo, EMBASE, Cochrane, Scopus, CINAHL, Web of Science, PEDro, and LILACS databases from August 8th to 14th, 2022, with an update in April 2023. There were no searches in the grey literature. Adjustments to the search strategy were made according to the specificities of each database, maintaining a similar combination of descriptors as stated in the registered protocol and Supplementary file 1.

### Eligibility criteria

Quantitative, cross-sectional, and longitudinal observational (descriptive and exploratory) and experimental studies of different methodological designs involving Brazilian children in early childhood (0 to 6 years old) in situations of social vulnerability were included. Only full articles published in Portuguese and/or English were considered. Studies without social vulnerability criteria, those involving foreign children, qualitative studies, studies on infant mortality, maternal care, letters, editorials, reviews, and those available only in abstract format were excluded.

### Study selection and data extraction

The study selection occurred in four stages: identification of articles in the databases, exclusion of duplicates using the Mendeley reference manager, reading titles and abstracts, and finally, reading the full texts with data stratification. The screening of studies was independently conducted by two reviewers (AMB and DOS). In cases of disagreement, a third reviewer (RLSM) was involved to decide on the inclusion or exclusion of some studies.

For data extraction, a form was developed and tested on the first five studies to assess its coherence with the research objective. Data extraction included: (1) study characteristics (authors, year of publication, title, language of publication, study type, study objectives, Brazilian regions, proponent institution, and access link); (2) journal metrics (journal title, impact factor, and indexing source); (3) sample characteristics (sample size and age range); (4) study area (grouped into 04 major health areas: Nutrition, Development, Health conditions, and Dentistry); (5) characterization of the situation of social vulnerability; and (6) outcomes presented in the study. For the analysis of journal impact factors, the Journal Citation Report (JCR) classification was considered.

## Results

Through searches conducted in the databases, 3162 studies were identified, with 1014 duplicate publications removed. After screening by title and abstract, 2039 studies were excluded, and 109 full texts were evaluated, resulting in 31 exclusions: 2 studies were not retrieved for full reading, and 29 did not meet the eligibility criteria. Thus, 76 articles were included for data extraction.^23^, ^24^, ^25^, ^26^, ^27^, ^28^, ^29^, ^30^, ^31^, ^32^, ^33^, ^34^, ^35^, ^36^, ^37^, ^38^, ^39^, ^40^, ^41^, ^42^, ^43^, ^44^, ^45^, ^46^, ^47^, ^48^, ^49^, ^50^, ^51^, ^52^, ^53^, ^54^, ^55^, ^56^, ^57^, ^58^, ^59^, ^60^, ^61^, ^62^, ^63^, ^64^, ^65^, ^66^, ^67^, ^68^, ^69^, ^70^, ^71^, ^72^, ^73^, ^74^, ^75^, ^76^, ^77^, ^78^, ^79^, ^80^, ^81^, ^82^, ^83^, ^84^, ^85^, ^86^, ^87^, ^88^, ^89^, ^90^, ^91^, ^92^, ^93^, ^94^, ^95^, ^96^, ^97^, ^98^ Supplementary file 2 illustrates the study selection process.

Table 1 presents the general characteristics of the studies. The first publication included in this scoping review dates back to 1984.^23^ A substantial increase in research involving early childhood social vulnerability is observed from the year 2000 until March 2023. During this period, 92.11% of the sample in this review was published,^26^, ^27^, ^28^, ^29^, ^30^, ^31^, ^32^, ^33^, ^34^, ^35^, ^36^, ^37^, ^38^, ^39^, ^40^, ^41^, ^42^, ^43^, ^44^, ^45^, ^46^, ^47^, ^48^, ^49^, ^50^, ^51^, ^52^, ^53^, ^54^, ^55^^,^^58^, ^59^, ^60^, ^61^, ^62^, ^63^, ^64^, ^65^, ^66^, ^67^, ^68^, ^69^, ^70^, ^71^, ^72^, ^73^, ^74^, ^75^, ^76^, ^77^, ^78^, ^79^, ^80^, ^81^, ^82^, ^83^, ^84^, ^85^, ^86^, ^87^, ^88^, ^89^, ^90^, ^91^, ^92^, ^93^, ^94^, ^95^, ^96^, ^97^, ^98^ with only 7.89% of publications from 1984 to 1999.^23^, ^24^, ^25^, ^26^^,^^55^^,^^56^ In the comparison between regions of the country, the Northeast region conducted the highest quantity of research,^26^^,^^44^^,^^45^^,^^48^^,^^49^^,^^52^^,^^54^^,^^56^, ^57^, ^58^, ^59^^,^^65^^,^^68^, ^69^, ^70^^,^^74^^,^^76^^,^^77^^,^^79^^,^^82^^,^^83^^,^^85^, ^86^, ^87^^,^^92^^,^ ^95^^,^^96^ followed by the Southeast region.^23^^,^^29^, ^30^, ^31^^,^^39^^,^^47^^,^^60^^,^^61^^,^^64^^,^^66^^,^ ^67^^,^^72^^,^^73^^,^^80^^,^^81^^,^^84^^,^^91^^,^^92^^,^^97^ Additionally, many studies are multicentric or have a national scope.^27^^,^^34^^,^^42^^,^^43^^,^^35^^,^^38^^,^^40^^,^^41^^,^ ^46^^,^^53^^,^^63^^,^^68^^,^^82^ No specific studies from the Central-West region were included. Federal public education institutions were responsible for over 70% of the studies,^23^^,^^25^^,^^28^^,^^30^, ^31^, ^32^, ^33^, ^34^, ^35^, ^36^, ^37^, ^38^, ^39^, ^40^, ^41^, ^42^, ^43^^,^^45^, ^46^, ^47^, ^48^, ^49^, ^50^, ^51^, ^52^^,^^59^^,^^61^, ^62^, ^63^, ^64^, ^65^^,^^67^^,^^70^^,^^71^^,^^74^^,^^76^, ^77^, ^78^^,^^80^, ^81^, ^82^, ^83^^,^^85^^,^^88^, ^89^, ^90^^,^^93^^,^^96^^,^^97^ followed by state public institutions,^27^^,^^29^^,^^44^^,^^54^^,^^60^^,^^66^^,^^72^^,^^73^^,^^84^^,^^87^^,^^91^ together accounting for more than 85% of the total sample. Foreign institutions^26^^,^ 56, 57, 58^,^^92^^,^^79^^,^^86^ represented a higher percentage of publications than private institutions^29^^,^^51^^,^^53^^,^^94^ in the country. Regarding language, English publications were prevalent, representing more than two-thirds of the total sample,^23^^,^^24^^,^^26^^,^^28^^,^^31^, ^32^, ^33^^,^^35^^,^^37^^,^^38^^,^^40^, ^41^, ^42^^,^^44^^,^^45^^,^^47^, ^48^, ^49^, ^50^^,^^52^^,^^55^, ^56^, ^57^, ^58^, ^59^, ^60^, ^61^^,^^63^, ^64^, ^65^, ^66^, ^67^, ^68^, ^69^, ^70^^,^^72^, ^73^, ^74^, ^75^, ^76^, ^77^^,^^79^^,^^81^^,^^83^, ^84^, ^85^, ^86^^,^^88^, ^89^, ^90^, ^91^, ^92^, ^93^^,^^95^^,^^98^ followed by publications in Portuguese.^27^^,^^33^^,^^34^^,^^39^^,^^41^^,^^43^^,^ ^51^^,^^54^^,^^62^^,^^78^^,^^82^^,^^87^^,^^96^

**Table 1:** General characteristics of the included studies.

Characteristics	n (%)
**DECADE OF PUBLICATION**	
1980–1989	2 (2.63)
1990–1999	4 (5.26)
2000–2009	12 (15.79)
2010–2019	37 (48.69)
2020–2023*	21 (27.63)
**BRAZILIAN REGION**	
North	01 (1.32)
South	12 (15.79)
Southeast	19 (25.00)
Northeast	30 (39.77)
Midwest	0 (0.0)
Multicentric studies (various regions)	14 (18.42)
**LANGUAGE**	
English	58 (76.31)
Portuguese	12 (15.79)
English/Portuguese	6 (7.90)
**PROPOSING INSTITUTIONS**	
Public Federal Institutions	54 (71.05)
Public State Institutions	11 (14.47)
Private Institutions	4 (5.26)
Foreign Institutions	7 (9.22)
	
**TOTAL**	**76 (100)**

⁎ Months from January to March.

In Table 2, the authors present the specific characteristics of the studies and the quality of the journals. A significant percentage of studies were cross-sectional and observational/exploratory in nature^26^^,^^33^^,^^54^ unlike experimental studies, which represented less than 3% of the sample.^76^ Regarding the quality and relevance of the journals, over 80% of the studies were published in journals with an impact factor^23^, ^24^, ^25^, ^26^^,^^32^ with more than two-thirds of these having an impact factor above 2. All eligible studies are in the Health field and were classified into four major sub-areas: Nutrition^23^, ^24^, ^25^, ^26^, ^27^, ^28^, ^29^, ^30^, ^31^, ^32^, ^33^, ^34^, ^35^, ^36^, ^37^, ^38^, ^39^, ^40^, ^41^, ^42^, ^43^, ^44^, ^45^, ^46^, ^47^, ^48^, ^49^, ^50^, ^51^, ^52^, ^53^, ^54^, ^55^ Health conditions,^56^, ^57^, ^58^, ^59^, ^60^, ^61^, ^62^, ^63^, ^64^, ^65^, ^66^, ^67^, ^68^, ^69^ development (socio-affective, cognitive/language, and motor),^70^, ^71^, ^72^, ^73^, ^74^, ^75^, ^76^, ^77^, ^78^, ^79^, ^80^, ^81^, ^82^ and Dentistry.^91^, ^92^, ^93^, ^94^, ^95^, ^96^, ^97^ It is essential to note that although classified in this way, researchers come from various fields, including medicine, nursing, nutrition, physiotherapy, psychology, biology, physical education, and biomedicine, among others.^83^, ^84^, ^85^, ^86^, ^87^, ^88^, ^89^, ^90^ It was observed that the majority of studies are in the field of Nutrition,^23^, ^24^, ^25^, ^26^, ^27^, ^28^, ^29^, ^30^, ^31^, ^32^, ^33^, ^34^, ^35^, ^36^, ^37^, ^38^, ^39^, ^40^, ^41^, ^42^, ^43^, ^44^, ^45^, ^46^, ^47^, ^48^, ^49^, ^50^, ^51^, ^52^, ^53^, ^54^, ^55^ a small part in Dentistry^91^, ^92^, ^93^, ^94^, ^95^, ^96^, ^97^ followed by those inter/multi-areas, meaning the investigated outcomes covered two or more health areas.^83^, ^84^, ^85^, ^86^, ^87^, ^88^, ^89^, ^90^ In Table 3, the authors present a synthesis of the main characteristics of social vulnerability, the main outcomes, and sample size by area of study. They were divided into four major areas: Nutrition, Health conditions, Development, and Dentistry. Studies that covered more than one area, i.e., inter/multi-areas, were exposed in this table, individually identifying each involved area. There is a more detailed breakdown of the extracted results in Supplementary materials 3 and 4.

**Table 2:** Specific characteristics of studies and quality of Journals.

CHARACTERISTICS	n (%)
STUDY TYPE	
Observational/Exploratory Cross-Sectional	43 (56.57)
Descriptive Cross-Sectional	17 (22.37)
Exploratory Longitudinal	12 (15.79)
Randomized Clinical Trial	2 (2.63)
Quasi-Experimental	1 (1.32)
Descriptive Longitudinal	1 (1.32)
	
STUDY AREA	
Nutrition	33 (43.42)
Health conditions	14 (18.42)
Development	13 (17.11)
Multi-Area	9 (11.84)
Dentistry	7 (9.21)
	
IMPACT FACTOR	
≥ 6	6 (7.89)
2 to 6	42 (55.27)
≤ 2	13 (17.10)
Without Impact Factor	15 (19.74)
TOTAL	**76 (100)**

**Table 3:** Summary of the main characteristics of social vulnerability and outcomes presented in the studies.

Study area and total sample number	Identification of studies 1st author and year of publication	Characterization of social vulnerability	Main outcomes presented in the studies
NUTRITION (*n* = 51.492)	Donangelo (1984);^23^ Victora et al. (1986);^24^ Post (1999);^25^ Souza et al. (1999);^26^ Silva et al. (2000);^27^ Post (2000)^28^ Nogueira et al. (2001);^29^ Oliveira et al. (2010);^30^ Domene et al. (2011);^31^ Ferreira et al. (2013);^32^ Ferreira et al. (2013);^33^ Santos (2013);^34^ Leite et al. (2013);^35^ Rauber et al. (2013);^36^ Buckstegge et al. (2014)^37^ Warkentin et al. (2013);^38^ Vega et al. (2014);^39^ Konstantyner et al. (2014);^40^ Poblacion et al. (2014);^41^ Rauber et al. (2014);^42^ Bortolini et al. (2015);^43^ Bezerra et al. (2015);^44^ Mayneris-perxachs et al. (2016);^45^ Lima et al.(2018);^46^ Rinaldi (2019);^47^ Lucena et al. (2019);^48^ Mendes et al. (2021);^49^ Silva et al. (2020);^50^ Rodrigues et al. (2020);^51^ Batista et al. (2020);^52^ Rebouças et al. (2020);^53^ Pedraza (2021);^54^ Mendes et al. (2021)^55^.	1- Brazilian Socioeconomic Classification Criteria of the Brazilian Association of Population Studies (ABEP); 2-PNAD indicators*(Family income; parents' education; number of children; type of housing; number of people and number of rooms per house; Water supply and sanitation; 3- Children in public daycare centers. 4- Marital status of the mother. 5- Beneficiaries of the Bolsa Família Program.	1- Food insecurity; Malnutrition and anemia associated with low maternal education and environmental variables, such as type of housing, degree of crowding and type of sewage disposal. 2- High levels of morbidity associated with low levels of parental education, low access to health services and precarious housing conditions. 3- Breastfeeding for > 4 months associated with reduced frequency of head circumference deficit. 4- Greater immunization coverage, growth monitoring and prenatal care associated with a lower prevalence of inadequate weight gain. 5- Guidance regarding the quality of the diet in early childhood tends to last throughout childhood. 6- Hypovitaminosis “A” associated with low maternal education and low birth weight. 7- Mothers under 20 years old and with low income associated with a higher risk of early weaning.
HEALTH CONDITIONS (*n* = 16.783)	Schorling et al. (1990);^56^ Agnew (1998);^57^ Newman et al. (2001);^58^ Barreto et al. (2006);^59^ Brandt et al. (2012);^60^ Lozer et al. (2013);^61^ Viana et al. (2013);^62^ Escobar et al.(2015);^63^ Roque et al. (2017);^64^ Ambrogi et al. (2021);^65^ Alves et al. (2020);^66^ Pina et al. (2020);^67^ Ferreira et al. (2021);^68^ Souza et al. (2022);^69^ Mitter et al. (2012);^86^	1- Brazilian Socioeconomic Classification Criteria of the Brazilian Association of Population Studies (ABEP); 2-PNAD indicators*(Family income; maternal education; number of children; type of housing; number of people per home; waste disposal, access to drinking water. 3-Places of residence such as residents of favelas, indigenous, riverside or quilombola areas and multi-family residences. 4- Income transfer resource, emergency aid, 5- Situation of food insecurity.	1-High rates of diarrheal disease in children up to 5 years of age. 2-High degree of financial dependence on income transfer programs. 3- Exposure to infections by parasites, viruses and bacteria that cause diseases such as diarrhea, zika, tuberculosis, gastritis and stomach inflammation. 4-Increased risk to the mental and emotional health of children and mothers. 5-Greater risk of diarrhea among younger children associated with lower maternal education and lower family socioeconomic level. 6- High hospitalization rates for diseases of the respiratory system and infectious and parasitic diseases, with indigenous children being the most affected. 7-High prevalence of intestinal parasites among indigenous and riverside children associated with low environmental conditions.
DEVELOPMENT (*n* = 13.059)	Santos et al. (2008);^70^ Lamy et al. (2011);^71^ Mello et al. (2014);^72^ Tella et al. (2018);^73^ Pacheco et al. (2018);^74^ Gonçalves et al. (2019);^75^ Silva et al. (2019);^76^ Correia et al. (2019);^77^ Delgado et al. (2020);^78^ Rocha et al. (2020);^79^ Souza et al. (2021);^80^ Morais et al. (2021);^81^ Munhoz et al. (2022)^82^	1-Social class determined by the Brazilian Socioeconomic Classification Criteria of the Brazilian Association of Population Studies (ABEP). 2- PNAD indicators* (Family income and father's income; Education of the mother and/or father; whether there is running water; Own internal or external bathroom; Number of rooms and number of residents per household; Employment status of the head of family, number of siblings). 3-Place of residence - subnormal agglomeration (favela) in the census sector. 4-Income transfer benefit, 5-Percentage of women aged 10 to 29 responsible for households 6- Presence of the father. 7- House construction material 8-Family purchasing power	1-Delay in cognitive function associated with poor socioeconomic conditions. 2-Approximate probability of 50% of developmental delay in children from low-income families. 3-Low maternal education, paternal absence, inadequate sanitary conditions at home and in the neighborhood, low birth weight and short stature associated with delayed neuromotor development. 4- Adverse childhood experiences associated with low scores in five domains of child development: communication, gross motor coordination, fine motor coordination, personal and social problem solving. 5-Greater risk of hospital admissions due to Community-Acquired Pneumonia in children related to social vulnerability and inequity in these areas, as well as the difficulty of Primary Health Care in monitoring these children. 6-The better the home and daycare environment, the better the child's cognitive development. 7- Indication of lower prevalence of delay in poor families who participated in CCT programs compared to those who were eligible but did not participate.
DENTISTRY (*n* = 18.728)	Chaves et al. (2007);^83^ Dini et al. (2000);^91^ Rodrigues (2001);^92^ Peres et al. (2003);^93^ Ferreira et al. (2007);^94^ Lima et al. *(*2016);^95^ Cangussu et al. (2016);^96^ Antunes et al. (2020)^97^	1-PNAD indicators (Family income; Education of the mother or guardian; Occupation of the head of the family; number of children, number of people per room, housing conditions, 2- Place of residence (living in a region considered poor).	1-Low socioeconomic status associated with a higher prevalence of tooth decay. 2-Children in daycare centers without guidelines for sugar consumption have a higher prevalence of cavities. 3-High rate of dental caries in children associated with low maternal education and low family income. 4-Infection and malnutrition during the period of tooth development, harmful to tooth enamel.
DENTISTRY AND NUTRITION (*n* = 1.018)	Oliveira et al. (2008)^84^	1-Family income, parents’ education and number of children, own home, overcrowding (number of people per room.	1-Association between nutritional, socioeconomic factors and tooth decay. 2-Underweight children and those with adverse socioeconomic conditions and mothers with less than 8 years of education more likely to have cavities, including severe tooth decay.
NUTRITION AND HEALTH CONDITIONS (*n* = 6.597)	Viana et al. (2013);^62^ Lima et al. (2010);^85^ Pedraza (2016);^87^ Marques et al. (2020);^88^ Murray et al. (2023)^90^	1-Brazilian Economic Classification Criteria (CCEB); 2- Place of Residence: - Residents in indigenous communities. 3- Type of Residence: - Canvas residence. 4- Beneficiaries of the Bolsa Família Program. 5- Family purchasing power. 6-Situation of food insecurity	1-Significant reduction in new parasitic infections, especially by Giardia spp, with the intervention of vitamin A. 2-Higher prevalence of children hospitalized for pneumonia or diarrhea in Food Insecure households. 3-Food insecurity; anemia; zinc deficiency; high frequency of health problems; eosinophilia; parasitism and poly parasitism. 4-Underweight and insufficient weight associated with smoking mothers, with less than 4 years of schooling, up to 24 months between births.
NUTRITION, HEALTH CONDITIONS AND DENTISTRY (*n* = 63)	Silveira et al. (2021)^89^	1-The study only states that they are children living in shelters for reasons such as: intra-family violence (physical or psychological), sexual abuse, sexual exploitation, neglect or abandonment.	1-The most prevalent disease was asthma, which is the main cause of hospitalization among Brazilian children and is positively associated with stress.

ABEP, Brazilian Association of Population Studies; CCEB, Brazilian Economic Classification Criteria; CCT, Collective Bargaining Agreement.

⁎ Indices from PNAD - IPEA are used to define the Social Vulnerability Index (SVI); 16 indicators are utilized: waste collection, water and sewage, commuting time to work, infant mortality, children aged 0 to 5 out of school, children aged 6 to 14 out of school, children who neither study nor work, low income, young mothers (10 to 17 years), mothers without elementary education and with more children up to 15 years old, illiteracy, children in households where no one has completed elementary education, income up to R$255 (half the minimum wage in 2010), low income and dependency on the elderly, unemployment, child labor, and informal employment without elementary education. ** Two studies did not specify the sample size: Nutrition (Silva et al., 2020) and Health Conditions (Souza et al., 1999).^27^

**Figure 1:**
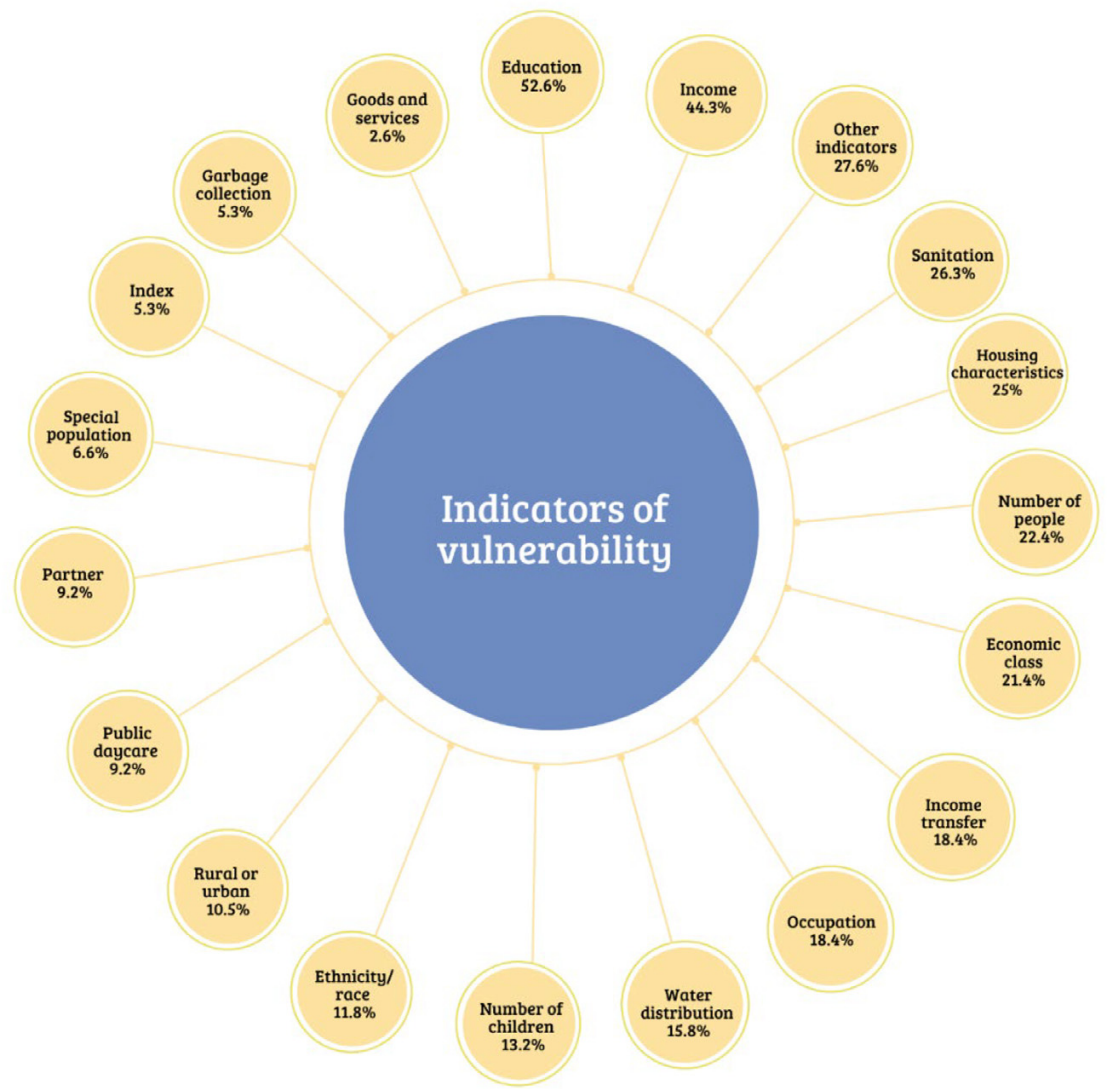
Vulnerability indicators used in studies.

In Figure 1, the detailed and percentage-based presentation of how vulnerability indicators were reported in the studies is shown. In many studies, more than one indicator was used to define the situation of social vulnerability, and therefore, the sum of the percentages expressed in the figure exceeds 100. There were cases where a composite index was created from various indicators.^47^^,^^67^^,^^70^^,^^81^ Education and income, both family and per capita, were the most commonly used indicators.^24^^,^^25^^,^^28^^,^^30^^,^^31^^,^^41^, ^42^, ^43^^,^^46^, ^47^, ^48^, ^49^, ^50^^,^^54^^,^^59^^,^^60^^,^^62^^,^^71^^,^ ^75^^,^^90^^,^^92^^,^^97^ Education, in most cases, referred to mothers, as did occupation.^30^^,^^32^^,^^35^^,^^36^^,^^37^^,^^40^^,^^43^^,^^44^^,^^51^^,^^62^^,^^72^^,^^79^^,^^82^^,^^91^^,^^96^ Household characteristics, such as the type of material used in its construction, the number of rooms, internal bathroom, independent kitchen, and services such as sanitation, treated water, and garbage collection, were also explored in the studies.^28^^,^^31^^,^^32^^,^^35^^,^^44^^,^^47^^,^^51^^,^^52^^,^^53^^,^^56^^,^^57^^,^^59^^,^^65^^,^^71^^,^^72^^,^^78^ Some studies only indicated the type of population, such as indigenous, *quilombola*, slum resident, or rural settlement resident, as a defining factor for socially vulnerable conditions.^37^^,^^58^^,^^61^^,^^64^^,^^68^^,^^74^^,^^83^^,^^85^^,^^86^ The least mentioned indicators, exposed in Figure 1 as "other indicators," included internet use,^53^ landline telephone,^59^ electricity,^72^ homeownership,^24^ mode of transportation, local violence,^78^ literacy rate for those over 20 years old,^46^ region of residence (North, South, Southeast, Northeast, Midwest), Gini index[93] and food insecurity.^29^^,^^45^^,^^46^^,^^62^^,^^63^^,^^90^

**Figure 2:**
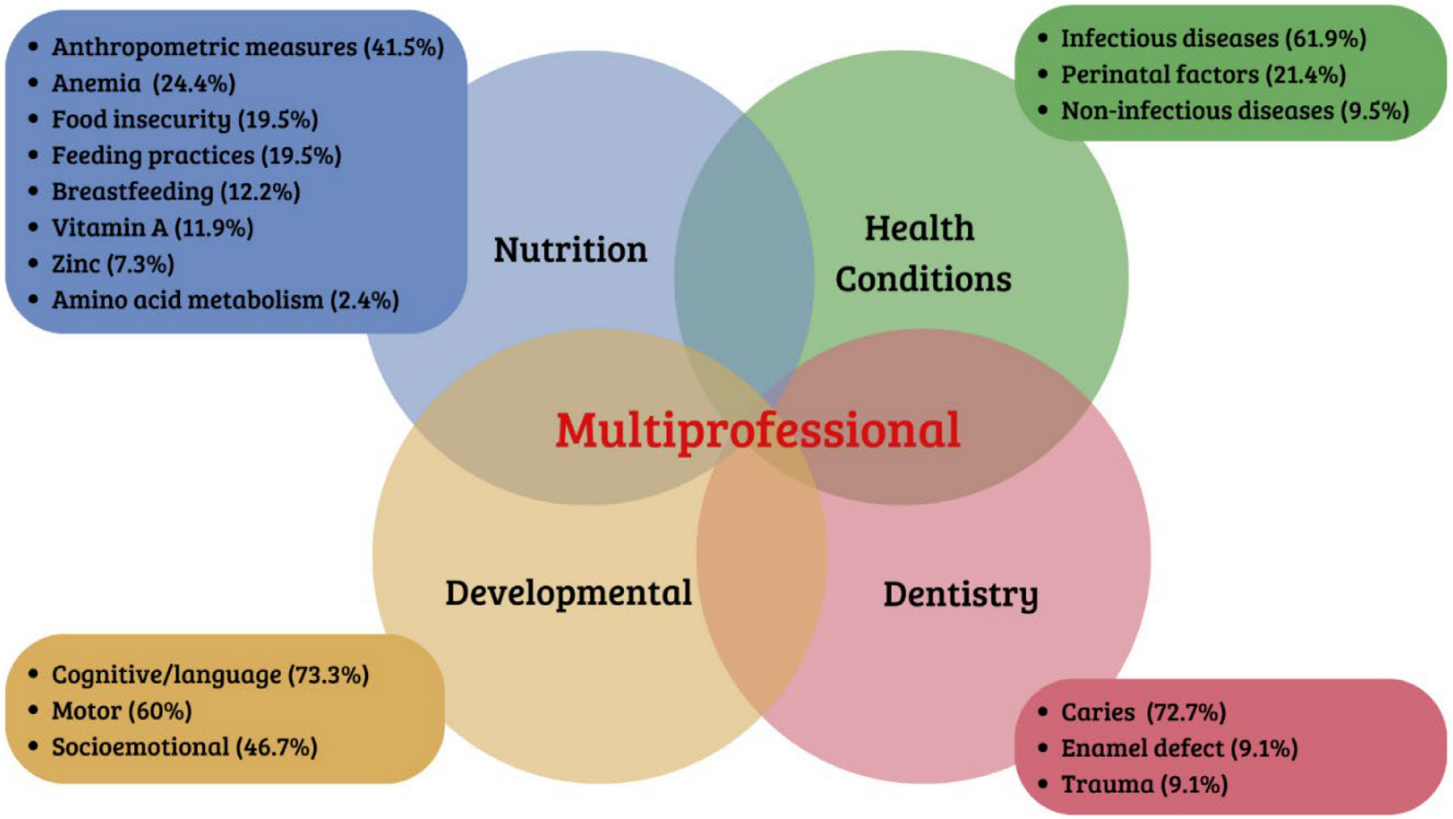
Main outcomes found in studies by area.

In Figure 2, a detailed breakdown of outcomes found by area is presented, considering the percentage within each area. It is important to note that a study in a particular area may have analyzed different outcomes. In Nutrition studies, anthropometric measurements are primarily evaluated to diagnose nutritional status,^23^, ^24^, ^25^, ^26^^,^^29^^,^^32^^,^^34^^,^^48^^,^^83^^,^^86^, ^87^, ^88^, ^89^ followed by dietary practices to assess diet quality and nutritional guidance, as well as breastfeeding and the presence of vitamins, amino acids, and minerals.^26^^,^^31^^,^^32^^,^^37^^,^^38^^,^^45^ Health conditions studies mainly assessed infectious diseases such as diarrhea, parasitic infections, pneumonia, COVID-19, tuberculosis, and the presence of Helicobacter pylori.^41^^,^^56^^,^^57^^,^^59^^,^^61^^,^^63^^,^^64^^,^^66^^,^ ^67^^,^^88^ These were followed by factors presented in the neonatal period such as low birth weight and health conditions during this period^58^^,^^60^^,^^62^^,^^68^^,^^69^^,^^83^^,^^98^ Some research^65^^,^^89^ also focused on non-infectious-diseases typical of this period, such as asthma. Studies involving Dentistry particularly evaluated the presence or prevention of dental caries,^84^^,^^89^^,^^91^, ^92^, ^93^, ^94^, ^95^, ^96^, ^97^ followed by trauma and defects in tooth enamel.^83^ In the Development area, the evaluation primarily involved cognitive or language development,^70^^,^^71^^,^^73^, ^74^, ^75^^,^^77^^,^^79^, ^80^, ^81^, ^82^ followed by motor development (gross and/or fine) and socio-emotional development.^71^^,^^75^, ^76^, ^77^^,^^79^^,^^82^

## Discussion

The 76 included studies presented relevant findings, including the temporal trajectory of eligible study publications, the variability of social vulnerability indicators, the association between vulnerability indicators and negative outcomes in early childhood, the scarcity of intervention studies, and the fact that 100% of eligible studies were in the field of Health.

In conducting searches across major databases without any temporal restrictions, the authors sought to expose how the Brazilian State has been involved with childhood issues throughout history and to determine how important guidelines are to maximize child health outcomes.^99^ The first eligible article meeting the inclusion criteria for this review was identified only in 1984, nearing the end of the 20th century. Until the end of the 1990s, only five more eligible studies were found, constituting 7.89% of the sample. These findings align with reports from authors such as Del Priore^100^ and Romagnoli,^101^ who, in addressing the history of childhood in Brazil, point to the invisibility of poor children in the country. Studies included from the 2000s onward represent 92.11% of the sample in this scoping review, coinciding with the expansion of public policies in the country. Additionally, Brazil is a signatory to important international treaties for early childhood development, such as the Convention on the Rights of the Child (CRC, 1989), the Universal Declaration of Human Rights (UDHR, 1948), and other international instruments for the defense of rights, such as the Millennium Development Goals (MDGs) and the Sustainable Development Goals (SDGs).^102^^,^^103^

It is important to highlight the nature of publications according to the national historical scenario. In the 1970s and 1980s, the country's concern was with infant mortality and malnutrition. For this reason, studies in the subsequent decades were mostly in the fields of nutrition and health conditions. With the improvement of living conditions in the country, better birth control, and the emergence of public policies such as the Family Health Strategy, Community Health Agents, and income transfer like the Family Allowance Program, there was a reduction in malnutrition and infant mortality rates. From that point on, studies related to early childhood development began to emerge.^104^ It is noteworthy that only from the year 2008 onwards does a focus on child development appear in the studies.

Another aspect observed is the lack of standardization and often the fragility of indicators characterizing the social vulnerability of families. Less than 25% of the studies used well-known indicators such as the Brazil Economic Classification Criterion (CCEB) produced by the Brazilian Association of Companies and Research (ABEP) or the São Paulo Social Vulnerability Index (IPVS) developed by the São Paulo State Data Analysis System Foundation (SEADE). Several studies used some items from the indicators of the National Household Sample Survey (PNAD). Other studies relied solely on places of residence or cohabitation, inferring a vulnerability condition, such as residents of slums, Indigenous communities, *quilombola* communities, rural areas, or those attending public daycare. Some studies only mentioned being a vulnerable population without presenting elements justifying such a condition. These findings are consistent with the literature, which reports a diversity of concepts or definitions of the term social vulnerability, among others.^6^^,^^7^ In other words, in Brazil, there is no precise conceptual definition, as well as standardization of indicators capable of characterizing the condition of social vulnerability for more aligned use in research.^7^

The low educational level of parents or guardians is often highlighted in many studies as an indicator of social vulnerability, with this indicator most commonly associated with maternal education. Indeed, this is one of the most consistent indicators to reflect conditions related to childhood.^81^^,^^82^ Studies consistently correlate low maternal education with negative outcomes in the lives of children, such as food insecurity, anemia, low birth weight, a higher incidence of dental caries, and worse rates of hospitalization for preventable causes, among others.^52^^,^^84^^,^^102^ Historically, Brazil maintained high illiteracy rates. In 1940, the Brazilian Institute of Geography and Statistics (IBGE) surveys indicated that 56.7% of the Brazilian population aged 10 or older was illiterate. In 1970, this rate was 32.9%, and by the year 2000, it reached 16.7% for the same age group.^105^ In this sense, it is essential to emphasize that education strongly influences the well-being of citizens. Higher levels of education are associated with longevity, better purchasing power, better health, and a lower likelihood of involvement in crime and violence.^79^

Regarding the outcomes and results of the studies, it was found that social vulnerability was consistently associated with worse outcomes. For example, food insecurity, malnutrition, and anemia appear as outcomes in a large part of the studies in the field of Nutrition. Similarly, infectious diseases in the field of health conditions and the presence of dental caries in Dentistry are highlighted. It is worth noting that interventions to combat malnutrition and infectious diseases in Brazil have reduced infant mortality and had an impact on improving other health conditions.^99^ However, there are still areas of vulnerability in the country, such as the North and Northeast, and some mesoregions in Minas Gerais state. It is necessary not only to provide greater care for these children but also to implement public policies for economic improvement in these regions, considering that children exposed to poverty and exclusion face a higher risk of perpetuating poverty through generations.^9^

Another observed result is that all eligible studies in this scoping review are from the health field. Considering that the searches were not directed to any specific area, it was expected to find studies from other fields, such as education and social protection. This is especially noteworthy because there is a considerable number of articles that mention the population directly linked to the Social Protection Policy, that is, beneficiaries of income transfer programs such as Bolsa Família and others.^39^^,^^44^^,^^48^, ^49^, ^50^^,^^54^^,^^55^^,^^65^^,^^75^^,^^77^^,^^79^^,^^90^ There is also within the scope of Social Protection the Comprehensive Family Care Program (PAIF) that benefits children in early childhood respect,^106^ but in the present research, no studies on this topic were mapped. The same consideration applies to the field of education, which serves this population in early childhood education institutions.

In 2016, the Ministry of Health published a guiding document aimed at promoting early childhood through multisectoral actions, which includes offering parenting education programs, actions focused on food and nutrition, access to daycare and preschool, home visits to support families, and promotion actions in Primary Health Care.^5^ In terms of national and international documents, there is recognition of the need for a joint intersectoral effort to promote the well-being of children in early childhood in situations of social vulnerability.^9^^,^^10^^,^^15^^,^^19^ A good example of an intersectoral public policy is the Family Allowance income transfer program, implemented by Social Protection but with conditionalities to be met by beneficiaries in the Health and Education sectors.^107^

The percentage of intervention studies included in this review is less than 5%. This finding highlights the need to develop more research with this type of design. Intervention studies have the potential to identify interventions that can truly produce effective results, thereby guiding governments in the planning of public policies. Only two experimental studies and one quasi-experimental study were included in this review. One of these studies assessed the impact of a public policy, specifically the Best Early Childhood Program from the state of Rio Grande do Sul. This program proposes intervention for early childhood development through home visits from pregnancy to six years of age.^75^ In a literature review, Batura et al. (2014)[105] analyzed intervention studies in early childhood in developed, medium-income, and low-income countries. The review discusses the importance of this type of study and emphasizes that multiple interventions, such as nutrition, stimulation, and family care performed simultaneously, can ensure better lifelong outcomes. However, it warns that it costs more, and concludes that more studies on the cost-effectiveness of these interventions would help low- and middle-income countries choose this type of intervention.^105^

It is essential to conduct evaluations of existing government policies and programs. There were no studies that assessed the impact of state policies on improving conditions in early childhood in the country, even though Brazil has long had important income transfer programs, such as the Family Allowance Program, the PAIF, and the Family Health Strategy, among others. The research conducted over the years has provided a situational diagnosis, but it is essential to indicate interventions capable of contributing to overcoming the challenges imposed by the situation of social vulnerability. Therefore, studies with experimental or intervention designs are crucial. The scarcity of intervention studies in early childhood in Brazil is one of the gaps identified in this Scoping Review. The lack of production of studies in the fields of Education and Social Protection related to socially vulnerable early childhood is also highlighted. It is recommended, therefore, to produce more intervention studies, preferably of an intersectoral nature, capable of measuring the effectiveness of investments in important public policies focused on the needs of early childhood in the country.

In this review, 100% of the studies were in the health sector. Social vulnerability is not an isolated condition; it encompasses various challenges such as access to basic goods and services. The health, education, and social assistance sectors comprise the three main public policies of the Brazilian state, each with principles and guidelines based on the guarantee of citizens' rights as a state responsibility. Therefore, the complex needs of children in social vulnerability cannot be fully addressed without collaboration between health, education, and social protection. No study involving all three sectors was found in this review, highlighting the need for future research to investigate the benefits of cooperation among these sectors for children in situations of social vulnerability.

It is worth noting that this review has limitations. Despite a considerable sample that included 76 studies, there may be eligible studies that did not appear in the searches, especially as publications in Spanish were not included. Grey literature was also not searched. Another aspect concerns the lack of studies in the areas of Social Protection and Education. Although the search was comprehensive, it is possible that some important terms or keywords were missed. On the other hand, this study contributes by presenting the state of the art regarding Brazilian children in early childhood in situations of social vulnerability. It also highlights some existing gaps and provides recommendations.

## Conclusion

This scoping review sought to verify what the country has achieved in terms of health, well-being, and child development in early Brazilian childhood and what studies reveal about this reality. The results revealed a significant increase in studies involving Brazilian children in early childhood in situations of social vulnerability over the last two decades. The presented outcomes underscored that social vulnerability is extremely detrimental to early childhood, exposing children to various forms of preventable illnesses. The mapping conducted also suggests a growing awareness in the country regarding the importance of investing in the care and protection of this developmental phase, aiming to ensure a better future for children. Moreover, it allows us to infer that both the scientific community and state policy interests are aligned in contributing to overcoming the effects of social vulnerability in early childhood in Brazil.

## Grant support

This study was funded by the Conselho Nacional de Desenvolvimento Científico e Tecnológico - CNPq (grant number 150010/2022-2).

## Conflicts of interest

The authors declare no conflicts of interest.

## Appendix Supplementary materials

Image, application 1

## Footnotes

Supplementary material associated with this article can be found in the online version at doi:10.1016/j.jped.2024.06.012.
