# Supplementary material for: Social vulnerability among Brazilian children in early childhood: a scoping review
Source: J Pediatr (Rio J). 2024 Aug 17;101(1):7–20. doi: 10.1016/j.jped.2024.06.012 (PMC11763581; doi:10.1016/j.jped.2024.06.012)
Supplement: Supplementary file 1 [file mmc1.docx]

**JPED-D-23-00506 – Supplementary Material**

**Supplementary File 1** Search strategy in databases.

| **SOURCE OF INFORMATION** | **SEARCH CONDUCTED** | **ITEMS FOUND** | **SEARCH DATE** |
| --- | --- | --- | --- |
| **MEDLINE**  **(PudMed)** | 1. “adverse experiences” 2. “biopsychosocial risk” 3. “social vulnerability” 4. Or\1-3 5. “Brazilian children” 6. “childhood in Brazil” 7. Or\5-6 8. “child development” 9. “early intervention” 10. “early stimulation” 11. “public policies” 12. “public health” 13. “maternal care” 14. “maternal depression” 15. “food and nutrition insecurity” 16. “support social” 17. “family dysfunction” 18. “nutritional status” 19. poverty 20. “mental disorders” 21. “child institutionalized” 22. “ domestic violence” 23. “sexual abuse” 24. “physical activity” 25. “child education” 26. kindergartens 27. “responsive care” 28. pre school 29. “public policies for children” 30. Or\8-29 31. 4 AND 7 AND 30 | 263 results | August 8, 2022 |
| **Scielo** | “adverse experiences” OR “biopsychosocial risk” OR “social vulnerability” OR “child development” OR “early intervention” OR “early stimulation” OR “public policies” OR “public health” OR “maternal care” OR “maternal depression” OR “food and nutrition insecurity” OR “support social” OR “family dysfunction” OR “nutritional status” OR “poverty” OR “mental disorders” OR “ Child institutionalized” OR “domestic violence” OR “sexual abuse” OR “domestic violence” OR “sexual abuse” OR “physical activity” OR “responsive care” OR “child education” OR “kindergartens” OR “preschool” OR “public  policies for children” | 534 results | August 8, 2022 |
| **Embase** | (adverse experiences OR biopsychosocial risk OR social vulnerability) AND (Brazilian children OR childhood in Brazil) AND (child development OR early intervention OR early stimulation OR public policies OR public health OR maternal care OR maternal depression OR food and nutrition insecurity OR support social OR family dysfunction OR nutritional status OR poverty OR mental disorders OR Child institutionalized OR domestic violence OR sexual abuse OR physical activity OR child education OR kindergartens OR responsive care OR pre school OR public policies for children) | 784 results | August 13, 2022 |
| **CINAHL** | (adverse experiences OR biopsychosocial risk OR social vulnerability) AND (Brazilian children OR childhood in Brazil) | 771 results | August 14, 2022 |
| **Web of Science** | ((adverse experiences OR biopsychosocial risk OR social vulnerability) AND (Brazilian children OR childhood in Brazil) AND (child development OR early intervention OR early stimulation OR public policies OR public health OR maternal care OR maternal depression OR food and nutrition insecurity OR support social OR family dysfunction OR nutritional status OR poverty OR mental disorders OR Child institutionalized OR domestic violence OR sexual abuse OR physical activity OR child education OR kindergartens OR responsive care OR pre school OR public policies for children)) | 278 results | August 14, 2022 |
| **LILACS (BVS)** | “adverse experiences” OR “biopsychosocial risk” OR “social vulnerability” OR “child development” OR “early intervention” OR “early stimulation” OR “public policies” OR “public health” OR “maternal care” OR “maternal depression” OR “food and nutrition insecurity” OR “support social” OR “family dysfunction” OR “nutritional status” OR “poverty” OR “mental disorders” OR “ Child institutionalized” OR “domestic violence” OR “sexual abuse” OR “domestic violence” OR “sexual abuse” OR “physical activity” OR “responsive care” OR “child education” OR “kindergartens” OR “pre school” OR “public  policies for children” | 124 results | August 13, 2022 |
| **Cochrane** | (education) OR (kindergartens) OR (responsive care) OR (preschool)adverse experiences) OR (biopsychosocial risk) OR (social vulnerability) AND (Brazilian children) OR (childhood in Brazil) AND (child development) OR (early intervention) OR (early stimulation) OR (public policies) OR (public health) OR (maternal care) OR (maternal depression) OR (food and nutrition insecurity) OR (support social) OR (family dysfunction) OR (nutritional status) OR (poverty) OR (mental disorders) OR (Child institutionalized) OR (domestic violence) OR (sexual abuse) OR (physical activity) OR (child | 56 results | August 13, 2022 |
| **Scopus** | ( ALL ( ‘adverse AND experiences’ OR ‘biop sychosocial AND risk’ OR ‘social AND vul nerability’ ) AND ALL ( ‘child AND develop ment’ OR ‘early AND intervention’ OR ‘ear ly AND stimulation’ OR ‘public AND polici es’ OR ‘public AND health’ OR ‘maternal  AND care’ OR ‘maternal AND depression’  OR ‘food AND nutrition AND insecurity’ O R ‘support AND social’ OR ‘family AND d dysfunction’ OR ‘nutritional AND status’ OR ‘poverty’ OR ‘mental AND disorders’ OR ‘ child AND institutionalized’ OR ‘domestic AND violence’ OR ‘sexual AND abuse’ OR ‘physical AND activity’ OR ‘child AND e ducation’ OR ‘kindergartens’ OR ‘responsive AND care’ OR ‘pre AND school’ ) ) | 170 results | August 12, 2022 |
| **PEDro** | 1. Abstract & Title: adverse experiences 2. Abstract & Title: biopsychosocial risk 3. Abstract & Title: social vulnerability | 31 results | August 12, 2022 |

**Supplementary Material 2** PRISMA flow chart diagram.

Articles identified through database searches (n=3,162)

CINAHL = 771

COCHRANE=56

EMBASE = 784

LILACS = 124

Medline/ PubMed = 414

Scielo=534

Scopus = 170

PEDro =31

Web of Science=278

**Identification**

Records removed before screening:

Duplicate records removed

(n = 1014)

Records screened for title and abstract (n =2.148 )

Reports assessed for eligibility (n =107)

Reports searched for recovery (n =109)

Reports excluded: (n=31)

●No social vulnerability criteria (n = 9)

●Age over 6 years old (n=8)

●Maternal care (n=2)

●Foreign children (n= 5)

●Infant mortality (n= 4)

●Qualitative Research (n=3 )

Records excluded (n =2.039 )

Reports not retrieved (n = 02)

**Screening**

Studies included in the review

(n = 76)

**Included**

## **Supplementary file 3** Included characteristics studies’ and periodic factor of impact.

| **1st Author (year); Brazilian State** | **Study objective** | **Study design** | **Sample**  **(n; age group)** | **Periodic Impact Factor **** |
| --- | --- | --- | --- | --- |
| **FIELD OF INTEREST- NUTRITION (n=33 )** | | | | |
| Donangelo et al. (1984);[23]  Rio de Janeiro | Characterize the nutritional status regarding zinc of children from low-income families. | Cross-sectional, exploratory | 103 Brazilian children aged 3 months to 6 years | **0.545** |
| Victora et al. (1986);[24]  Rio Grande do Sul | Estimate the prevalence of malnutrition in children aged 12-35.9 months. | Cross-sectional, exploratory | 802 children aged 12 to 35.9 months | **13.831** |
| Post et al. (1999);[25]  Rio Grande do Sul | Investigate the relationship between abdominal circumference and weight for height in children, compared among four groups: Brazilian stunted and non-stunted, Peruvian, and North American. | Cross-sectional, descriptive and exploratory | 386 children, age 6 to 59 months | **2.772** |
| Souza et al. (1999);[26]  Ceará | Evaluate the relationship between health services, socioeconomic variables, and inadequate weight gain in Brazilian children: infants (0-11 months) and children (12-23 months) in 140 municipalities in the State of Ceará. | Cross-sectional, exploratory | Infants (0-11 months) and children (12-23 months) in 140 municipalities in the State of Ceará | **13.831** |
| Silva et al. (2000);[27]  São Paulo | Describe the access of Brazilian children to daycare centers in different macroregions, focusing on their nutritional status and considering their income level. | Cross-sectional, descriptive | 10,667 children under 6 years | **1.141** |
| Post et al. (2000);[28]  São Paulo | Investigate variations in body proportions, particularly in abdominal circumference. Half of the children belonged to a high socioeconomic status (SES) group, and the other half were from low-income families. | Cross-sectional, descriptive | 197 children aged 12 to 35.9 months | **0.545** |
| Nogueira et al. (2001);[29]  São Paulo | Characterize the nutritional status of these children, mainly through anthropometric data, and relate it to their hematological status. | Cross-sectional, exploratory | 115 institutionalized children aged 12 to 72 months in daycare centers in the municipality of Pontal. | **0.545** |
| Oliveira et al. (2010);[30]  São Paulo | Document the prevalence of short stature, overweight, and anemia in preschoolers and examine whether these nutritional problems are related; also identify if these nutritional problems have the same risk factors. | Cross-sectional, exploratory | 270 children aged 4 to 29 months | **0.545** |
| Domene et al. (2011);[31]  São Paulo | Describe breastfeeding practices in vulnerable groups residing in the Northwest District of Campinas. | Cross-sectional, descriptive | 1,139 children up to six years of age | **1.141** |
| Ferreira et al. (2013);[32]  Alagoas | Investigate the effect of exclusive breastfeeding on the head circumference (HC) of children living in deprived/Quilombola communities.  PC - Perímetro Céfálico | Cross-sectional, exploratory | 725 children (365 boys and 360 girls) aged 12 to 60 months | **2.335** |
| Ferreira et al*.* (2013);[33]  Alagoas | Identify factors associated with vitamin A deficiency in children from the semi-arid region of Alagoas. | Cross-sectional, exploratory | 551 children under five years. | **JCR**  **Not found** |
| Santos et al. (2013);[34]  Rio Grande do Sul | Verify the relationship between food insecurity and the nutritional status of Brazilian children. | Cross-sectional, exploratory | 4,817 children under 5 years | **JCR**  **Not found** |
| Leite et al. (2013);[35]  Multicenter (National Coverage) | Evaluate the prevalence of anemia and associated factors among indigenous children in Brazil. | Cross-sectional, exploratory | 6,125 Indigenous childre <5 years of age | **4.344** |
| Rauber et al. (2013);[36]  Rio Grande do Sul | Evaluate the long-term effect of an intervention on the diet quality of school-age children and examine the tracking of food intake throughout childhood. | Longitudinal exploratory | 345 children assessed at 3-4 years of age and 307 children at 7-8 years of age | **4.125** |
| Buckstegge et al. (2014);[37]  Paraná | Evaluate factors associated with weaning in southern Brazilian children residing in low-income households. | Cross-sectional, exploratory | 124 mothers of children aged 1 to 12 months | **JCR**  **Not found** |
| Warkentin et. al. (2013);[38]  Multicenter (National Coverage) | Describe the duration and identify determinants of exclusive breastfeeding. | Cross-sectional, descriptive and exploratory | 1,704 children aged 0 to 24 months. | **1.141** |
| Vega et al. (2014);[39]  São Paulo | Characterize children under 2 years old beneficiaries of any current Conditional Cash Transfer Program (PCTR) in Brazil in 2006, with a focus on the Bolsa Família Program, regarding demographic and socioeconomic variables, as well as evaluate some nutritional variables, comparing them with the non-beneficiary population, exploring the achievement levels of the programs' objectives.  PRTC – Programa de Transferência Condicionada de Renda; | Cross-sectional, descriptive | 1,735 children aged 0-2 years. | **1.917** |
| Konstantyner et al. (2014);[40]  Multicenter (National Coverage) | Identify and quantify risk factors for vitamin A deficiency in a probabilistic sample of children under 2 years participating in a national survey in Brazil and provide a comprehensive model of risk factors to support health strategies and policies. | Cross-sectional, exploratory | Data from 1,436 children under 2 years of age from the National Survey of Demography and Health of Women and Children in 2006 | **2.244** |
| Poblacion et al. (2014);[41]  Multicenter (National Coverage) | Study the process of food insecurity and hunger in Brazilian households with children under five years old. | Cross-sectional, descriptive | 3,920 households (with 01 child under 5 years per household) | **3.371** |
| Rauber et al. (2014);[42]  Rio Grande do Sul | Evaluate the adaptation of the Healthy Eating Index (HEI) to Brazilian dietary recommendations for 3 to 4-year-old children.  Índice de Alimentação Saudável (IAS) | Longitudinal exploratory | Children aged 3 to 4 years (n = 345) and 7 to 8 years (n = 307) | **3.571**  Pediodic online page |
| Bortolini et al. (2015);[43]  Multicenter (National Coverage) | Evaluate the dietary practices of Brazilian children and the factors associated with diet quality and diversity. | Cross-sectional, exploratory | Data from 2,477 children aged 6 to 36 months from the National Survey of Demography and Health conducted in Brazil in 2006/2007 | **3.371** |
| Bezerra et al. (2015);[44]  Paraíba | Evaluate the (in)security of food situations for families with children under five residing in socially vulnerable areas and its association with biological characteristics, the health status of children, and family socioeconomic context. | Cross-sectional, exploratory | 76 families with children under five and with a former waste picker member from the deactivated landfill in Campina Grande, Paraíba | **1.141** |
| Mayneris-Perxachs et al. (2016);[45]  Ceará | Report a potential mechanistic role for altered choline and tryptophan metabolism in the effects associated with malnutrition and a potential role for increased nicotinamide N-methyltransferase (NNMT) in growth recovery with N-methylnicotinamide serving as a biomarker for this adaptation.  nicotinamida N-metiltransferase (NNMT) | Case-control study  Longitudinal exploratory | 326 children; 6.2–25.9 months of age. | **4.996** |
| Lima et al. (2018);[46]  Multicenter (National Coverage) | Analyze variables associated with vitamin A deficiency (VAD) in Brazilian children aged 6 to 59 months, considering a hierarchical model of determination.  VAD - vitamin A deficiency | Cross-sectional, exploratory | 3,417 children aged 06 to 59 months | **JCR**  **Not found** |
| Rinaldi et al. (2019);[47]  Alagoas | Evaluate the dietary pattern by socioeconomic gradient of Brazilian infants and young children in 2006 and 2013. | Longitudinal descriptive, and exploratory | 1,904 (0-1 year and 1 to 4 years) in 2006 and 4,215 in 2013. | **2.772** |
| Lucena et al. (2019);[48]  Alagoas | Evaluate the association between weight status and food insecurity in socially vulnerable children benefiting from a food assistance program (FAP) called the Milk Program, which aims to stimulate family farming and promote food access to needy families.  FAP -Programa de assistência alimentar - denominado Programa Leite | Cross-sectional, exploratory | 1,487 children aged 24 to 96 months | **4.539** |
| Mendes et al. (2022);[49]  São Paulo | Evaluate the association between iron deficiency anemia and complementary feeding in children under 2 years assisted by the Family Allowance Program. | Cross-sectional, exploratory | 1,604 children, aged 6 to 24 months | **4.539** |
| Silva et al. (2020);[50]  Paraná | Evaluate the variability of hemoglobin levels in children from socially vulnerable municipalities and its association with individual and municipal factors. | Cross-sectional, exploratory | Data from children (12 to 59 months) collected in 48 municipalities in the Southern region of Brazil, included in the Brazil Without Poverty Plan. | **3.371** |
| Rodrigues et al. (2020);[51]  São Paulo | Evaluate the food security situation of families with preschool children in the rural area of a municipality in the Vale do Paraíba region of São Paulo. | Cross-sectional, descriptive and exploratory | 98 mothers of children from four months to six years of age | **JCR**  **Not found** |
| Batista et al. (2020);[52]  Piauí | Evaluate the prevalence of anemia and its social determinants in Brazilian children from rural settlements in agrarian reform projects in the city of Teresina, Northeast Brazil. | Exploratory population-based cross-sectional study | 131 children under 5 years (rural settlement) | **JCR**  **Not found** |
| Rebouças et al. (2020);[53]  Multicenter (National Coverage) | Identify demographic characteristics, access to health services, and housing conditions related to the feeding practices of Brazilian children aged 12 to 23 months. | Cross-sectional descriptive analytical | 2,541 Brazilian children aged 12 to 23 months | **JCR**  **Not found** |
| Pedraza et al. (2021);[54]  Paraíba | Evaluate the food and nutritional insecurity situation of families with children under five residing in municipalities in the Metropolitan Region of João Pessoa. | Cross-sectional, descriptive | 321 children between 25 and 60 months | **1.917** |
| Mendes et al. (2021);[55]  São Paulo | Verify the association between socioeconomic and demographic characteristics and the dietary pattern (DP) of children served by the Family Allowance Program.  PD - padrão alimentar | Cross-sectional, exploratory | 1,604 children aged 6 to 24 months assisted by the (PBF) | **4.539** |
| **FIELD OF INTEREST-** **HEALTH CONDITIONS (n=14)** | | | | |
| Schorling et al. (1990);[56]  Ceará | Elucidate the occurrence of diarrheal diseases in children up to five years of age in an urban slum in northeastern Brazil. | Longitudinal exploratory | 175 children under 5 years of age | **5.363** |
| Agnew et al. (1998);[57]  Ceará | Examine the interaction of cryptosporidiosis and nutrition, comparing anthropometric indicators in 18 case-matched children and controls. | Case-control study | 154 children born between May 1989 and April 1993. | **7.759** |
| Newman et al. (2001);[58]  Ceará | Evaluate the epidemiology of Giardia lamblia infection and investigate factors associated with clinical manifestations and recurrence, examining the role of copathogens in the course of the disease. | Longitudinal exploratory | 157 children aged 4 years | **3.918** |
| Barreto et al. (2006);[59]  Bahia | Investigate the occurrence of diarrhea in 'sentinel areas' of Salvador, Brazil, and establish a preliminary profile of the most common pathogens present in childhood diarrhea through screening of fecal samples. | Longitudinal exploratory | nvestigated 1,057 children aged 0-3 years. | **Não localizado JCR** |
| Brandt et al. (2012);[60]  São Paulo | Study the development of fecal microbiota in a group of exclusively breastfed newborns living in low socioeconomic conditions in São Paulo, Brazil, during the first month of life. | Longitudinal Descriptive | 10 newborns up to 30 days | **6.876** |
| Lozer et al. (2013);[61]  Espírito Santo | Evaluate the prevalence of different categories of E. coli in symptomatic and asymptomatic children from low socioeconomic rural communities in southeastern Brazil. | Cross-sectional, exploratory | 141 children with diarrhea (<10 years) and 419 apparently healthy controls | **3.667** |
| Viana et al. (2013);[62]  Multicenter (National Coverage) | Update previous estimates of birth weight surveys and analyze social, economic, demographic, and epidemiological factors associated with insufficient weight and low birth weight in Brazilian children under two years. | Cross-sectional, exploratory | 1,916 children up to 24 months of age. | **3.371** |
| Escobar et.al. (2015);[63]  Multicenter (National Coverage) | Analyze the prevalence of diarrhea and associated factors in indigenous children under 5 years in Brazil. | Cross-sectional, exploratory | 5,828 indigenous children under 5 years. | **4.135** |
| Roque et al. (2017);[64]  São Paulo | Evaluate factors associated with Helicobacter pylori infection in children up to five years of age in an urban indigenous community of the Guarani ethnicity in São Paulo, Brazil. | Cross-sectional, exploratory | 74 children (age range 0.4-4.9 years) | **1.838** |
| Ambrogi et al. (2021);[65]  Alagoas | Describe and analyze the vulnerabilities of women and children most affected by the Zika epidemic in Brazil. | Cross-sectional, exploratory | 54 women with children affected by Zika. | **3.371** |
| Alves et al. (2020);[66]  São Paulo | Identify risk areas for the occurrence of tuberculosis in children and its association with social inequalities in a municipality in the southeast region of Brazil. | Cross-sectional, exploratory | 98 children under 15 years. | **2.567** |
| Pina et al. (2020);[67]  Santa Catarina | Map and assess the spatial risk of hospitalization for Community-Acquired Pneumonia in children under 5 years of age and its association with vulnerable areas. | Cross-sectional, exploratory | 265 hospitalized children under 5 years diagnosed with Community-Acquired Pneumonia | **2.567** |
| Ferreira et al. (2021);[68] Multicêntrico (Abrangência Nacional) | Analyze the birth weight of indigenous children from data from the I National Survey of Health and Nutrition of Indigenous People of Brazil. | Cross-sectional, descritive | 6,128 indigenous children under 5 years. | **3.371** |
| Souza et al. (2022);[69]  Bahia | Identify the incidence and spatial distribution pattern of children with SCZ in the municipality of Salvador, according to living conditions.  **SCZ** - Síndrome Congênita associada à infecção pelo vírus Zika | Cross-sectional, exploratory | 726 children born between August 1, 2015, and July 31, 2016, with reported microcephaly and SCZ. | **4.135** |
| **FIELD OF INTEREST- DEVELOPMENTAL (n= 13 )** | | | | |
| Santos et al. (2008);[70]  Bahia | Examine the impact of poverty on cognitive scores at five years of age, decomposing the possible causal pathways through which poverty affects cognition. | Longitudinal exploratory | 346 children aged 5 years | **4.135** |
| Lamy et al. (2011);[71]  Maranhão | Verify the prevalence of developmental delay diagnosis in 2-year-old children in an underprivileged community in São Luís, and its relationship with elements of the home environment. | Cross-sectional, exploratory | 184 children aged 2 years | **1.917** |
| Mello et al*.* (2014);[72]  São Paulo | Evaluate the use of Alberta Infant Motor Scale (AIMS) as an assessment tool for early detection of abnormalities.  AIMS - Alberta Infant Motor Scale (Escala Motora Infantil de Alberta) | Cross-sectional, descritive | 71 infants under 18 months of age, eutrophic, and at low biological risk at birth. | **Não localizado JCR** |
| Tella et al. (2018);[73]  São Paulo | Investigate the influence of socioeconomic level (SES) and maternal education on the linguistic, motor, and cognitive development of babies.  SES- socioeconomic status (NSE- Nível Sócio econômico ) | Cross-sectional, exploratory | 444 infants aged 6 to 9 months | **Não localizado JCR** |
| Pacheco et al. (2018);[74]  Maranhão | Explore potential differences in fluid intelligence and language between low birth weight and adequate birth weight children in a low-income neighborhood in São Luiz, Maranhão, Brazil, and to determine if potential effects can be mediated by environmental factors. | Cross-sectional, exploratory | 100 children, 53 with low birth weight and 47 with adequate birth weight | **Não localizado JCR** |
| Gonçalves et al. (2019);[75]  Rio Grande do Sul | Identify family and child characteristics associated with health outcomes in the development of 4 to 6-year-old children who participated in "First Better Childhood" (PIM).  PIM - Primeira Infância Melhor | Quasi-experimental | 571 children aged 4 to 6 years from eight cities in the South | **3.371** |
| Silva et al. (2019);[76]  Pernambuco | Investigate the consequences of exposure to VPI for the mental health of a child.  VPI – Violência por Parceiro Íntimo | Longitudinal exploratory | 614 mother-child pairs in a poor urban neighborhood in Recife, northeast Brazil | **4.863** |
| Correia et al. (2019);[77]  Ceará | Evaluate the prevalence of developmental delay in children and identify socioeconomic determinants. | Cross-sectional  observational | 3,566 children aged 2 to 72 months | **3.752** |
| Delgado et al. (2020);[78]  Rio Grande do Sul | Evaluate the motor development of children from four to 17 months and investigate its association with sociodemographic risk factors. | Cross-sectional, exploratory | 110 children aged four to 17 months | **Não localizado JCR** |
| Rocha et al. (2020);[79]  Ceará | Evaluate the association of MMH, IPV, and ACEs with scores of child communication, gross motor, fine motor, problem-solving, and personal-social development.  MMH – Maternal mental health (saúde mental materna); VPI - intimate partner violence (Violência por Parceiro Íntimo) e ACEs - Adverse childhood experiences, (Experiências Adversas na Infância); | Cross-sectional, exploratory | 3,566 children aged 0 to 66 months | **6.604** |
| Souza et al. (2021);[80]  São Paulo | Investigate possible effects of socioeconomic level on the performance of selective trust in Brazilian children. | Cross-sectional, exploratory | 93 children (3 to 6 years old) from two socioeconomic levels (43 from middle-class and 50 from low-SES families). | **2.58** |
| Morais et al. (2021);[81]  Minas Gerais | Investigate how socioeconomic factors and the quality of ecological environments affect the motor and cognitive development of economically disadvantaged children. | Cross-sectional, predictive, and correlational study. | 147 children aged 24 to 36 months. | **2.671** |
| Munhoz et al. (2022);[82]  Multicenter (National Coverage) | Evaluate socioeconomic, family, and individual factors associated with infant development in the first year of life among families in social vulnerability. | Cross-sectional, exploratory | 3,242 children < 12 months of age | **3.371** |
| **FIELD OF INTEREST- MULTIPROFESSIONAL (n= 09)** | | | | |
| Chaves et al. (2007);[83]  Paraíba | Evaluate the cumulative incidence of enamel defects (DE) and their correlation with life course events, such as malnutrition and pre- and post-natal infections, in a cohort of children with low socioeconomic status.  DE -defeitos de esmalte | Longitudinal exploratory | 246 children examined at 12 months of age and 228 at 36 months of age. | **1.33** |
| Oliveira et al. (2008);[84]  São Paulo | Assess if the dental caries status was related to the nutritional status in urban Brazilian preschool children aged 12 to 59 months. | Cross-sectional, exploratory | 1,018 children aged 12 to 59 months | **2.16** |
| Lima et al. (2010);[85]  Ceará | Evaluate the effects of retinol on intestinal barrier function, growth, total parasites, and Giardia spp. infections in children from Northeast Brazil. | Randomized double-blind placebo-controlled clinical trial | 79 children aged 2 months to 9 years. | **3.288** |
| Mitter et al. (2012);[86]  Ceará | Examine if apolipoprotein E4 affects intestinal barrier function, thus improving short-term growth and long-term cognitive outcomes in children from Brazilian slum. | Experimental/  Randomized Trial | 213 children aged 2 months to 9 years in a favela in Fortaleza/CE | **6.876** |
| Pedraza et al. (2016);[87]  Paraíba | Characterize the nutritional and morbidity profile of children attending public daycare centers in the municipality of Campina Grande, Paraíba. | Cross-sectional, descritive | 299 preschool-aged children | **3.371** |
| Marques et al. (2020);[88]  Rondônia | Evaluate intestinal parasitosis and nutritional status (anemia and linear growth) in preschoolers living in contemporary Amazonian communities. | Cross-sectional, exploratory | 937 preschool-aged children (1 to 59 months) from traditional (247) and immigrant mineira (688) families | **2.791** |
| Silveira et al. (2021);[89]  Pernambuco | Evaluate the association of stress exposure with social, clinical, and nutritional characteristics in children living in shelters in the city of Fortaleza. | Cross-sectional, exploratory | 63 children aged one to 60 months residing in shelters in a city in Northeast Brazil. | **2.567** |
| Murray et al. (2023);[90]  Rio Grande do Sul | Examine experiences that may increase the risk of mental health problems among caregivers and young children during the COVID-19 pandemic and the inequalities generated by pre-pandemic disadvantage. | Longitudinal exploratory | 2,083 children and caregivers with data from before the pandemic in 2019, when the children were 4 years old, and again in 2020. | **13.081** |
| FIELD OF INTEREST- DENTISTRY (n= 07 ) | | | | |
| Dini et al. (2000);[91]  São Paulo | Investigate the relationships between sociodemographic factors, children's dietary habits, oral hygiene, and the prevalence and patterns of caries in Brazilian children aged 3 to 4 years. | Cross-sectional, exploratory | 245 preschoolers aged 3 to 4 years. | **Não localizado JCR** |
| Rodrigues et al. (2001);[92] Pernambuco | Investigate the effects of dietary guidelines on sugar and sugar intake in daycares, and other potential risk factors for dental caries in two groups of low socioeconomic school-aged children. | Longitudinal exploratory | 510 3-year-old children from low-income families. | **3.264** |
| Peres et al*.* (2003);[93]  São Paulo | Investigate the association between dental caries in children in the state of São Paulo, Brazil, and socioeconomic development indices at the municipal level. | Cross-sectional, exploratory | 15,385 records of oral exams from 5 or 6-year-old children in 129 municipalities in the state of São Paulo. | **Não localizado JCR** |
| Ferreira et al. (2007);[94]  Rio Grande do Sul | Investigate the prevalence and severity of dental caries and its association with demographic and socioeconomic variables in Brazilian preschoolers. | Cross-sectional, exploratory | 1,487 children aged 0 to 5 years. | **3.264** |
| Lima et al. (2016);[95]  Piauí | Evaluate the prevalence of dental caries, dental brushing habits, fluoride intake, and factors associated with toothpaste use in Brazilian children according to socioeconomic status (SES).  SES- socioeconomic status (NSE- Nível Sócio econômico ) | Cross-sectional, exploratory | 148 children aged three to four years from low and high socioeconomic status. | **2.378** |
| Cangussu et al. (2016);[96]  Bahia | Identify factors associated with the incidence of caries in early childhood. | Cross-sectional, exploratory | 495 children aged 4 to 30 months. | **Não localizado JCR** |
| Antunes et al. (2020);[97]  Rio de Janeiro | Use clinical and socio-odontological indicators to assess the profile of dental trauma (TDI) in low-income Brazilian preschoolers.  TDI – Traumatismo Dentário | Cross-sectional, exploratory | 606 children aged 2 to 6 years. | **5.711** |

Brazilian states: Acre - AC; Alagoas - AL; Amapá - AP; Amazonas - AM; Bahia - BA; Ceará - CE; Espírito Santo - ES; Goiás - GO; Maranhão - MA; Mato Grosso - MT; Mato Grosso do Sul - MS; Minas Gerais - MG; Pará - PA; Paraíba - PB; Paraná - PR; Pernambuco - PE; Piauí - PI; Roraima - RR; Rondônia - RO; Rio de Janeiro - RJ; Rio Grande do Norte - RN; Rio Grande do Sul - RS; Santa Catarina - SC; São Paulo - SP; Sergipe - SE; Tocantins – TO.

** Impact Factor according to the Journal Citation Reports (JCR). Source: <https://impactfactorforjournal.com/jcr-impact-factor-2022/>

# Multicenter Studies - National Scope: involved a population from all States of the Federation."

**Supplementary file 4** Description of social vulnerability indicators and outcomes addressed by the study area of interest.

| **1st Author (year)** | **Social vulnerability indicator used in the study** | **Outcomes addressed in the study** |
| --- | --- | --- |
| **FIELD OF INTEREST- NUTRITION (n=33)** | | |
| Donangelo et al **(1984)[**23] | The study only mentions: "Low-income children according to verbal information from the caregiver." | According to the adopted criteria, 77.7% of children were classified as normal, 11.7% with first-degree malnutrition, and 10.7% with more severe malnutrition. A decrease in serum zinc in children was observed in relation to the degree of malnutrition. |
| Victora et al., **(1986)[**24] | Family income, parents' education, number of children, home ownership, overcrowding (number of people and number of rooms), type of housing, degree of overcrowding, and type of sewage disposal. | Among the social variables studied, family income and father's education were the two risk factors with the strongest associations with nutritional status. Environmental variables, particularly housing type, overcrowding, and sewage disposal type, were also strongly associated with malnutrition. There was a significant variation in malnutrition based on family income, with prevalence of growth delay, low weight, and weight loss being approximately 7, 4, and 2 times higher, respectively, among children in the poorest households compared to those in wealthier households. |
| Post et al. **(1999)[**25] | Family income, low parental education, inadequate home conditions, and the high prevalence of mothers with low height. | High levels of morbidity, low parental education levels, limited access to health services, and poor housing conditions. Stunted Brazilian children showed lower anthropometric measures compared to non-stunted and North American children. |
| Souza et al.,  **(1999)[**26] | The data refer to municipalities: Water supply and sanitation; family income; illiteracy rate; per capita GDP; urbanization percentage. | Inadequate weight gain was observed in 28.1% of infants and 34.9% of young children. Higher immunization coverage, growth monitoring, and prenatal care were associated with a lower prevalence of inadequate weight gain in infants and children. Female illiteracy rate and the percentage of households with low income were associated with inadequate weight gain. |
| Silva et al.,  **(2000)[**27] | Three income strata intervals were distinguished: ≤ US$40, US$40-80, and ≥ US$80. The children were also stratified according to the frequency of attendance at daycares that provided free meals. | For children under two years old, only 3.6% attended daycare. However, the proportion of older children (above five years) with access to daycare reached 28.22% (Table 1). The results reveal a significant difference, indicating that a substantial proportion of children, considered biologically more vulnerable, are excluded from this type of benefit. |
| Post **(2001)[**28] | Living conditions, caregiver's education. | Most indices were significantly lower among children in the low socioeconomic status (NSE) area. The most pronounced differences (>8% in relative terms) were observed for skinfold thickness, weight, and mid-upper arm areas (muscle, fat, and total). Significant differences ranging from 4 to 8% were also observed for subscapular height, total height, and seated height or crown-rump length, and for arm circumference. The differences were not significant for chest and abdominal circumferences or for the three skinfold thicknesses (triceps, suprailiac, and subscapular). These findings suggest that the use of North American standards to assess weight for height in Latin American populations deserves further evaluation, as it may lead to an underestimation of the true prevalence of underweight.  SES- socioeconomic status (NSE- Nível Sócio econômico ) |
| Nogueira et al.  **(2001)[**29] | Malnutrition. Low economic level but not measured. Children from public daycares. | The studied population revealed a peculiar aspect, namely the coexistence of good anthropometric nutritional status and iron-deficiency anemia. Micronutrient deficiencies, such as iron, were prevalent. |
| Oliveira et al. **(2010)[**30] | Mother's occupation, house quality, home goods and facilities, water and sanitation. | The results showed a high prevalence of overweight (22.2%), risk of stunting (22.6%), and anemia (37%). The percentage of daycare attendance, age, number of siblings under 5, and per capita income were associated with hemoglobin levels. |
| Domene et al.  **(2011)[**31] | Caregiver's education, monthly income, sewage destination, and water treatment. | Breastfeeding occurred in 93.3% of cases, with a median breastfeeding duration of 8 months (95% CI 7.0 - 8.9 months), and exclusive breastfeeding for a median of 4 months (95% CI 3.9 - 4.1 months). More than 20% of children received other foods in the first month. A significant portion of families reported not receiving guidance on breastfeeding in basic health units. |
| Ferreira et al. **(2013)[**32] | The socioeconomic level was determined through the classification of the Brazilian Association of Population Studies (ABEP).  ABEP - Associação Brasileira de Estudos Populacionais | The prevalence of length deficit was 13.3% among children breastfed exclusively for less than 30 days, 10.6% among those breastfed exclusively for 30 to 119 days, and 5.8% among those breastfed exclusively for 120 days or more. Even after controlling for possible confounding variables, exclusive breastfeeding for ≥ 4 months reduced the risk of length deficit (prevalence ratio, 0.48; 95% CI 0.24, 0.99). |
| Ferreira et al. **(2013)[**33] | Low weight, mothers with low education, number of family members, income supplementation program, income, whether the mother has a partner, sewage, piped water, water used for drinking, type of material used in house construction, number of rooms, rural or urban. | The prevalence of vitamin A deficiency was 45.4%, and the variables that remained associated after multivariable analysis were low maternal education (PR=1.66; 95% CI: 1.12-2.44), low birth weight (PR=1.41; 95% CI: 1.01-1.98), and age between 12.1 to 24 months (PR=1.45; 95% CI: 1.04-2.02). |
| Santos et al.  **(2013)[**34] | Sociodemographic variables (gender, macro-region of residence, domicile situation, household income quartiles, mother's age, mother's skin color, and child's age). | Out of 4,817 children, 7% had stunting, 7% were overweight, and 47% experienced food insecurity. The mean scores for height-for-age, weight-for-age, and weight-for-height were -0.31, 0.12, and 0.40, respectively, and were lower in children with food insecurity. |
| Leite et al.  **(2013)[**35] | Mother's education, family socioeconomic level, sanitation conditions, maternal anemia, and anthropometric deficits. | Hemoglobin levels were assessed in 5,397 children (88.1% of the total sample). The overall prevalence of anemia was 51.2%. There was a higher risk of anemia for boys, lower maternal education, lower family socioeconomic status, poorer sanitary conditions, presence of maternal anemia, and anthropometric deficits. Regional differences were observed, with the highest rate being observed in the Northern region. |
| Rauber et al. **(2013)[**36] | Socioeconomic characteristics analyzed were maternal education and family income. | Two main findings: 1st - the positive effect that dietary counseling during the first year of life had on diet quality at 3-4 years of age was lost by 7-8 years of age; 2nd - diet quality remains consistent during childhood, as the total HEI score did not change from preschool to school age in the control group.  HEI - Healthy Eating Index (IAS- Índice de Alimentação Saudável ) |
| Buckstegge et al., **(2014)[**37] | Weaning, maternal education, occupation, number of children. Children followed by the Pastoral. | The mean duration of breastfeeding was 5.6 months (SD = 4.0), with a median of 5 months. The cumulative conditional probability of breastfeeding until 6 months of age was 54.9%, and until 12 months, it was 34.2%. The results of this study showed that the use of bottles and pacifiers was related to a shorter duration of breastfeeding in these communities. |
| Warkentin et al., **(2013)[**38] | The socioeconomic class of families was determined by the Brazilian Economic Classification Criterion of the Brazilian Association of Population Studies (ABEP).  ABEP - Associação Brasileira de Estudos Populacionais | The use of a pacifier, mothers under 20 years of age, non-residence in the southeast region, and belonging to a lower economic class independently increased the risk of early weaning by 53%, 28%, 22%, and 28%, respectively. The median estimated duration of exclusive breastfeeding was 60 days. The final Cox model consisted of mother's age <20 years (hazard ratio = 1.53, 95% confidence interval = 1.11-1.48), pacifier use (hazard ratio = 1.53, 95% confidence interval = 1.37-1.71), not residing in the southeast region of the country (hazard ratio=1.22, 95% confidence interval=1.07-1.40), and socioeconomic status (risk ratio=1.28, 95% confidence interval=1.06-1.55). |
| Vega et al.,  **(2014)[**39] | Brazilian Economic Classification Criterion (CCEB), 2008 version, which quantifies eight durable household goods and the number of monthly employees, in addition to the education level of the head of the family, generating cutoff points for determining the purchasing power of the person or family. The higher the score, the higher the purchasing power. From such information, the economic condition for the household is estimated. For this study, the eight strata suggested in the original proposal of the cutoff points of the Brazilian Association of Research Companies (2007/2008) were aggregated into five (A, B, C, D, and E).  CCEB - Brazilian Economic Classification Criterion (Critério de Classificação Econômica Brasileiro CCEB) | Most children under 2 years old in families benefiting from PTCR in 2006 had characteristics corresponding to the most vulnerable population, such as residing in poorer regions of the country, having lower family income, having lower purchasing power, having lower maternal education, and being in food insecurity, reaching the population groups for which they were created. There is a high income inequality in Brazil, with the Northeast Region being the most unequal and the South Region being the least unequal. There is a high degree of financial dependence on the value transferred by income transfer programs, especially among families in the Northeast region. |
| Konstantyner et al. **(2014)[**40] | Mother's education and income. | The prevalence of vitamin A deficiency was estimated at 16.1% (95% CI, 12.7 to 20.2). |
| Poblacion et al. **(2014)[**41] | To assess the economic condition, the Brazil Economic Classification Criterion (CCEB) was used, which quantifies eight durable goods in the household, the number of monthly employees, and the educational level of the head of the family, generating cutoff points for determining the purchasing power of the individual or family. | The study found a high prevalence of moderate and severe food insecurity in the North and Northeast regions in 2006 (30.7%), in economic classes D and E (34%), and in recipients of Income Transfer Programs (ITP; 36.5%). |
| Rauber et al.  **(2014)[**42] | Maternal education and family income were considered. | The mean HEI score was 65.7 ± 11.2 at 3-4 years and 65.0 ± 8.8 at 7-8 years. The IAS was positively correlated with food variety and food groups (cereals, vegetables, fruits, and meats/beans), except for milk at 3-4 years, and negatively with sodium, total fat, and saturated fat intake. The HEI score was moderately to strongly associated with dietary fiber and various micronutrients. |
| Bortolini et al., **(2015)[**43] | Marital status, mother's education, occupation, economic level. | Children from less privileged socioeconomic classes and those living in households with severe food insecurity had approximately 40% less chance of having a high-quality diet. The chance of having a diversified diet was 71% lower for children living in households with severe food insecurity and 43% lower if they were daughters of mothers with low education. Children living in the North Region of the country had fewer chances of having a diversified and high-quality diet. The diet quality of Brazilian children is inadequate, and social vulnerability is strongly associated with this unfavorable dietary situation. |
| Bezerra et al. **(2015)[**44] | Mother's education; number of rooms in the home; waste destination in the home; feces destination in the home; number of people in the home; participation in the Family Allowance Program; per capita household income in minimum wages. | The prevalence of food insecurity characterized 96.1% of families, with moderate forms (34.2%) and severe forms (32.4%) predominating. Families in which there were children who had lost weight in the last 15 days and those with uncollected garbage had higher chances of moderate food insecurity and severe food insecurity, by 9.49 times (95% CI=1.95-46.26) and 6.71 times (95% CI=1.34-33.59), respectively. |
| Mayneris-  Perxachs et al. **(2016)[**45] | Not specified, it only says they are from Ceará and in a state of malnutrition. | Malnutrition disrupts various biochemical pathways, including choline and tryptophan metabolism, and increases the proteolytic activity of the intestinal microbiome. Additionally, metabolic adaptation was observed in malnourished children to reduce energy expenditure, N-methylnicotinamide, and reduced excretion of β-aminoisobutyric acid. |
| Lima et al. **(2018)[**46] | Economic class (A to E) according to the Brazilian Association of Research Companies: per capita income; mother's age and education; food insecurity. | The prevalence of vitamin A deficiency (DVA) was 17.5%, with a higher prevalence in the Southeast and Northeast regions. The mother's years of education variable did not show a significant association with the occurrence of DVA; however, it was observed that children whose mothers had less than five years of education were more likely to have DVA. Moreover, a significant number of children with DVA were found in severe food insecurity, but no significant association was found between the analyzed variables.  DVA - deficiência de vitamina A |
| Rinaldi et al.  **(2019**)[47] | The wealth index was calculated based on information about the goods available in households, sewerage, and the education level of the head of the family. | Breastfeeding was more common for poor infants and young children, while the consumption of fresh foods (fruits, vegetables, meat, beans) was higher among the wealthy in 2006 and 2013. Biscuits and sweetened beverages were more consumed by infants and wealthy young children in 2006 and by poor and wealthy children in 2013. |
| Lucena et al., **(2019)[**48] | Socioeconomic variables were: child's gender; education (illiterate/literate) and work situation (employed/unemployed) of the adult responsible for the household; and household crowding index (i.e., the number of residents divided by the number of rooms in the home). These socioeconomic data were collected through a pre-tested questionnaire. | Out of 376 children (25.3%), 164 (11.0%) were overweight, and only twenty-seven (1.8%) were underweight. Seventy-six percent of families experienced some degree of food insecurity. Multivariable analysis did not reveal a general association between household food insecurity and weight status. In specific comparison, children living in severe food insecurity were less likely to be obese than children living in food security (prevalence ratio = 0.60; 95% CI 0.38, 0.96; P = 0.03). |
| Mendes et al. **(2021**)[49] | Housing characteristics: per capita household income; number of residents per household; location of residence (urban/rural); source of drinking water (adequate: mineral or public/inadequate: well water, river, or cistern) (23); sanitation (adequate: general sewerage system or septic tank/inadequate: rudimentary septic tank or open sewer). | Among the children, 58.1% had dietary diversity, and 9.7% were stunted. Approximately 40% of households had inadequate sanitation, 41.5% of caregivers had less than 9 years of education, and 66.5% of evaluated households had mild, moderate, or severe food insecurity. |
| Silva et al. **(2020)[**50] | Monthly family income per capita, based on a minimum wage (MW) in 2015 (R$ 788.00/US$ 236.63), number of residents in the household, mother/guardian's occupation, enrollment in daycare or kindergarten, Beneficiary of the Family Allowance Program. | Lower hemoglobin levels were observed in children residing in municipalities with higher urbanization rates and a lower number of Community Health Agents. At the individual level, lower hemoglobin values were identified for children under 24 months not enrolled in daycare, beneficiaries of the Family Allowance Program, and diagnosed with low weight. |
| Rodrigues et al. (**2020)[**51] | Maternal/Family Sociodemographic Characteristics: Marital status, mother's occupation and education, social benefit, type of water and sewerage in the residence. | Fifty-one percent of families were in a situation of food insecurity, with the mild form prevailing. An association was found between food insecurity and variables such as maternal education, family income, and receipt of social benefits. Associations were observed between food insecurity and maternal education (p=0.023), family income (p=0.000), and receipt of social benefits (p=0.035). |
| Batista et al.  **(2020**)[52] | Social Determinants: Family size (number of people), maternal education, paternal education, per capita income, government support, type of housing, number of people per room, public garbage collection. | The prevalence of anemia was 29%. Poisson regression analysis showed that the prevalence of anemia decreased by 39% for each year of the child (aPR=0.61; 95% CI=0.50 - 0.74), 14% for each year of maternal education (aPR=0.86; 95% CI=0.79 - 0.94), and 6% for each year of maternal age (aPR=0.94; 95% CI=0.89 - 1.00). Additionally, children living in houses made of unfinished mud or masonry had a higher prevalence of anemia than those living in finished masonry houses (aPR=2.73; 95% CI=1.50 - 4.97). |
| Rebouças et al. **(2020)[**53] | Housing Conditions: Number of residents per room, occupant's condition in the house, predominant material of house walls, water supply to the residence, water used for drinking, residence with a kitchen, residence with a television, residence with a microwave, residence with internet access. | The results of this study suggest that the country's region and area of residence, skin color, internet access, water used for drinking, and the number of residents per room were commonly associated with inadequate dietary practices that do not meet the Ministry of Health's recommendation to consume foods from different food groups daily. Children residing in the North or Northeast regions, especially in rural areas, non-white children, and those who had their last medical consultation more than a year ago were more likely to exhibit inadequate dietary practices. Despite 65% having their growth and development monitored in public health institutions, only half of the households receive visits from community health agents, and 8.3% receive no form of follow-up. |
| Pedraza et al. **(2016)[**54] | The characteristics of family socioeconomic context evaluated were: Maternal education, maternal work outside the home, number of people in the household, household sanitation, Family Allowence Program benefit, family per capita income | Sixty-three point nine percent of families experienced some degree of food and nutritional insecurity, mainly in moderate/severe forms (44.6%). Severe food and nutritional insecurity affected 15.0% of households. |
| Mendes et al. **(2021)[**55] | The caregivers of the children were interviewed through a structured questionnaire covering sociodemographic, health, and environmental issues. Information on family income, sanitation, residence location, and the number of residents per household was obtained from secondary data through the Consultation, Selection, and Extraction of Information Database of the Single Registry for Social Programs of the Brazilian government (Cadastro Único), using the social registration number (NIS) of each individual provided by the state government. | This study identified an association between socioeconomic inequities and early dietary patterns, with the early introduction of inappropriate complementary feeding practices in children served by the Family Allowance Program in the state of Alagoas. The influence of sociodemographic characteristics, particularly maternal age, and low caregiver education, in shaping the early dietary pattern of children served by the Family Allowance Program in the state of Alagoas. |
| **FIELD OF INTEREST** HEALTH CONDITIONS **(n=14)** | | |
| Schorling et al. **(1990)[**56] | Approximately one-third of the houses were temporary "squatter" dwellings made of clay and wood, and two-thirds were permanent, built of brick and adobe. The main sources of drinking water were two public taps, open for several hours a day. Less than 20 percent of the houses had running water or indoor sanitation facilities. | The diarrhea attack rates were very high across all age groups. Overall, these children experienced more than 11 episodes of diarrhea per year, spending an average of almost 82 days per year with diarrheal illness. |
| Agnew et al.  **(1998)[**57] | Approximately one-third of the houses were temporary "posseiro" dwellings made of clay and wood, and two-thirds were permanent dwellings built of brick and adobe. The main sources of drinking water were two public taps, two outdoor communal sinks open for several hours a day, and two surface wells. Less than one-third of the houses had running water or indoor sanitation facilities. | The majority of children under 2 years from families benefiting from PTCR in 2006 had characteristics corresponding to the most vulnerable population, such as residing in poorer regions of the country, having lower family income, lower purchasing power, lower maternal education, and experiencing food insecurity, affecting the population groups for which they were created. There is significant income inequality in Brazil, with the Northeast region being the most unequal and the South region being the least unequal. There is a high degree of financial dependence on the value transferred by income transfer programs, especially among families in the Northeast region.  PRTC – Programa de Transferência Condicionada de Renda; |
| Newman et al**. (2001)[**58] | Children from a slum in Fortaleza, low socioeconomic status, low weight for age. | Of the 157 children followed for 3 months or more, 43 (27.4%) were infected with Giardia. The organism was identified in 8.8% of all stool samples and, although found at a similar frequency in non-diarrheal stools (7.4%) and diarrheal stools (9.7%), it was more common in children with persistent diarrhea (20.6%) than acute diarrhea (7.6%, P=0.002). Recurrent or reinfection was common (46%). Children with symptomatic infections had significantly lower weight-for-age and height-for-age than asymptomatic children. Copathogens were not associated with the course of the disease. |
| Barreto et al.  **(2006)[**59] | Socioeconomic and sanitary conditions, landline or cell phone, independent kitchen, dirt floor at home, caregiver with less than 4 years of education. | Viral and bacterial pathogens were associated with episodes of severe diarrhea, while viral and protozoal pathogens were associated with longer episodes. |
| Brandt et al.  **(2012)[**60] | The parents had low education and low income; the families lived in multifamily homes, and some of them lived in slums. These communities had electricity and running water, although access to water varied in each house. The sewage system was inadequate. | The results demonstrate that the microbiota of newborns changed from a simple bacterial profile to a more complex one. The high degree of colonization by enterobacteria observed during the first month of life in these Brazilian neonates was consistent with findings in other studies. The change in colonization may have occurred as a result of being born in developing countries and is possibly related to a high degree of exposure and environmental contamination. |
| Lozer et al.  **(2013)[**61] | Quilombola population. | Diarrheagenic E. coli strains were isolated from 253 (45.2%) children and were associated with diarrhea in children under 5 years old (p < 0.001). EAEC (20.9%), DAEC (11.6%), EPEC (9.3%) were the most frequent pathotypes, followed by ETEC (2.7%), EIEC (0.5%), and STEC (0.2%). Depending on the assay, EPEC, EAEC, and DAEC strains (collectively referred to as enteroadherent E. coli) were isolated in 45% to 56% of diarrhea cases, a significantly higher incidence than in controls (P < 0.05). Individually, only DAEC showed a significant association with diarrhea (p < 0.05), especially in children aged 2 to 5 years.  DEC - Diarrheagenic E. Coli- (Cepas diarreiogênicas de E. Coli)  EPEC -Enteropathogenic E. coli (EPEC -E. coli enteropatogênica)  ETEC - enterotoxigenic E. coli (ETEC -E. coli enterotoxigênica  EIEC - enteroinvasive E. coli ( EIEC - E. coli enteroinvasiva),  EHEC - enterohemorrhagic E. coli (EHEC - enterohemorrágica E. coli)  STEC - Shiga-toxin producing E. coli (STEC - E. coli produtora de toxina Shiga),  EAEC - Enteroaggregative E. coli (EAEC - E. coli enteroagregativa ),  DAEC -Diffusely adherent E.coli (DAEC - E. coli difusamente aderente) |
| Viana et al.  **(2013)[**62] | Family purchasing power (A1-C1 and C2-E) 17, maternal education (< 4 years and ≥ 4 years), food insecurity. | Low birth weight and insufficient weight were associated with smoking mothers, mothers with less than 4 years of schooling, primiparous women, women with an interpregnancy interval of less than 24 months, and mothers who did not want to have children when they became pregnant. |
| Escobar et al.  **(2015**)[63] | Maternal education, lower family socioeconomic level, malnutrition, house construction material, number of people at home, waste disposal, drinking water. | Information on diarrhea was obtained for 5,828 children (95.1% of the total sample). The overall prevalence of diarrhea was 23.5%. Regional differences were observed, with a higher rate in the Northern region (38.1%). Higher risk of diarrhea was observed among younger children and those with lower maternal education, lower family socioeconomic status, malnutrition (weight deficit for age), the presence of another child with diarrhea in the household, and the occurrence of upper respiratory tract infection. |
| Roque et al.  **(2017)[**64] | Indigenous community. | High prevalence in individuals over three years of age, suggesting that the incidence of infection was higher in the first three years of life. Previous antibiotic use was inversely associated with current Helicobacter pylori infection. |
| Ambrogi et al. **(2021)[**65] | Income transfer, ethnicity, income, number of children, education, sewage, garbage collection, type of housing. | In most visited municipalities, there was no public transportation to either of the two state reference centers for pediatric early stimulation therapy. More than half of the women (55%, n = 27) were entirely dependent on municipal-provided transportation to take their children to weekly 30-minute physical therapy/occupational therapy/early stimulation sessions. On average, the round trip to the services took 3 hours.45% (n = 22) reported that, due to the lack of transportation services provided by the municipality, they could not take their children to early stimulation sessions. The most remote families traveled up to 6 hours round trip for a 30-minute pediatric early stimulation therapy session. |
| Alves et al.  **(2020)[**66] | Age, neighborhood of residence. Geoprocessed area of social vulnerability (Paulista Social Vulnerability Index - income among other indicators that are not clear). | There were 96 cases of childhood tuberculosis, of which 90 were geocoded through a process of converting addresses into geographical coordinates. An area of risk was identified in the municipality, where children under 15 have a 3.14 times higher risk of contracting tuberculosis than those living outside this area. Tuberculosis remains a problem linked to living conditions, as it is significantly and persistently influenced by socioeconomic and cultural factors that exacerbate indices of inequalities and social injustices. |
| Pina et al.  **(2020)[**67] | Social vulnerability was investigated through the Paulista Social Vulnerability Index (IPVS) Inventory prepared by the State Data Analysis System (SEADE) Foundation and based on information derived from the 2010 Brazilian Demographic Census. This index takes into account variables such as per capita household income, the percentage of women aged 10 to 29 responsible for households, and the situation of a subnormal cluster (slum) in the census tract. | The findings revealed geospatial locations with a higher risk of hospitalizations for Community-Acquired Pneumonia in children related to social vulnerability and inequity in these areas, as well as the difficulty of Primary Health Care in monitoring these children. |
| Ferreira et al.  **(2021)[**68] | Residents in Indigenous Communities. | Records of birth weight were not found for 26.7% of the 6,128 children sampled in the documents searched. The North region had the highest number of children without birth weight data (51%), while the other regions had less than 30% missing data, ranging from 18.3% (Northeast) to 26.7% (South/Southeast). The average birth weight of the 3,994 analyzed children (65.2% of the total sample) was 3,201g (SD ± 18.6g), regardless of gender, type of delivery, and natality. The prevalence of low birth weight was 7.6% (n = 302). Low birth weight ranged from 7.3% in the Northeast to 7.9% in the Center. |
| Souza et al  **(2022)[**69] | Income calculation considered the proportion of heads of households with an average monthly income ≤ two minimum wages. Education considered the proportion of literate people aged 10 to 14. Sanitation considered the percentage of households connected to the general water supply. Slum considered the percentage of households in a subnormal cluster (slum). Inhabitants per room considered the average number of residents per household in relation to the average number of rooms used as bedrooms. | Seven hundred and twenty-six live births were reported, of which 236 (32.5%) were confirmed for Zika Virus Congenital Syndrome (ZCS). Despite reports of ZIKV infection being widely distributed, cases of ZCS were concentrated in poor areas of the city. A positive spatial association was observed between residing in locations with worse living conditions and the birth of children with ZCS.  ZCS Zika Virus Congenital Syndrome - **SCZ** - Síndrome Congênita associada à infecção pelo vírus Zika |
| Mitter et al.  **(2012)[**86] | The study does not specify how poverty or social vulnerability was characterized. It only mentions that the participants are residents of a slum. | The results of the present study indicate that APOE4-positive children receiving glutamine supplementation, in particular, showed improved short-term gains in HAZ (height-for-age), WAZ (weight-for-age), and WHZ (weight-for-height), which were correlated with better performance in long-term cognitive tests.  HAZ (height-for-age), WAZ (weight-for-age), and WHZ (weight-for-height) (HAZ - altura para idade; WAZ -peso para idade; WHZ - peso para idade) |
| Murray et al.  **(2023)[**90] | Family income, emergency aid, food insecurity. | Low-income families and black/mixed-race mothers in this study were at a much higher risk of severe financial losses, food scarcity, and children without contact with school or organized schools. These difficulties were associated with increased conflict in adult relationships, problems with parents, and children's concerns about food availability during the pandemic. In turn, these experiences were associated with an increase in mental health problems in children (conduct, emotional, and hyperactivity-inattention) and their caregivers (depression and anxiety). |
| **FIELD OF INTEREST – DEVELOPMENTAL (n=13)** | | |
| Santos et al.  **(2008)[**70] | Socioeconomic status: material resources (family purchasing power, family income, and father's income); family status (mother's literacy, father's presence, family type, religion). | Cognitive function at five years of age was negatively associated with poor socioeconomic conditions, low maternal education, paternal absence, inadequate sanitary conditions at home and in the neighborhood, low birth weight, and short stature; and positively associated with high levels of home stimulation and preschool attendance. |
| Lamy et al.  (2011)[71] | 50% of families with income equal to or less than 1 minimum wage, only 5.1% of mothers with more than 8 years of education, and a paternal unemployment rate of 27.3% or more. Housing and sanitation conditions also indicate daily difficulties, as 100% of families did not have piped water, and 69.9% did not have a toilet in their homes. | The prevalence of suspected developmental delay in these children, according to the Gesell scale assessment, was 44.9%, with adaptive behavior (56.8%) and language being the most affected areas. |
| Mello et al.  **(2014)[**72] | Maternal education (years of study), house construction material, piped water, electricity, private internal bathroom, private external bathroom, number of rooms, number of residents per household. | This study presented the highest prevalence of neuromotor delay when compared to the AIMS reference population. 8.5% of infants with atypical motor development, 7.0% classified as suspected, and 84.5 in the typical category. Namely: atypical motor development (Amd), suspected (Smd), and typical (Tmd).  AIMS - Alberta Infant Motor Scale (Escala Motora Infantil de Alberta)  Amd - atypical motor development (dmA- desenvolvimento motor Atípico);  Smd- suspected motor development ( mdS – desenvolviemnto motor suspeito)  Tmd - typical motor development - (mdT -desenvolvimento motor Típico);  mdT motor development – motor desempenho típico |
| Tella et al.  **(2018)[**73] | The socioeconomic status (SES) was assessed using the Brazilian Economic Classification Criteria proposed by the Brazilian Association of Research Companies (ABEP), which combines parents' education and the existence of consumer goods (as an estimate of purchasing power). This index provides an SES classification based on five levels: A, B, C, D, and E (A being the highest and E the lowest)  SES- socioeconomic status (NSE- Nível Sócio econômico )  ABEP - Associação Brasileira de Estudos Populacionais | Children with high socioeconomic status showed better performance in language and motor tasks, and more years of maternal education were related to higher scores in language and cognition. We found a low prevalence of significant developmental delay at 6 months of age in this cohort of births from the outskirts of São Paulo. Children from lower-income families showed an approximate 50% probability of developmental delay, even after adjusting for maternal education. |
| Pacheco et al.  **(2018)[**74] | Low-income neighborhood. No specific indicator was provided, only that "The socioeconomic backgrounds and household environments of the children were also explored through a caregiver questionnaire (Brazilian Child Environment Questionnaire).". | The results indicated significant differences between groups in fluid intelligence but not in language. The low birth weight group demonstrated poor fluid intelligence. Difficulties related to all reading tests, suggesting a delay in reading development. Environmental factors partially explained the results. Variables 'years of preschool' and 'number of educational games/books at home' predicted better test results. |
| Gonçalves et al. **(2019)[**75] | Level of education and occupation of both parents, number of siblings, and family income. | Among PIM children, lower family income, program exit time, city, and younger age were associated with a higher risk of developmental vulnerability and/or lower average scores in EDI domains.  PIM - Primeira Infância Melhor  EDI *- Instrumento de Desarrollo Temprano* (EDI -*Instrumento de Desenvolvimento na Primeira Infância* ) |
| Silva et al.  **(2019)[**76] | Socioeconomic variables: family income and father's education, mother's education, employment status of the head of the family, number of siblings. | 60.6% of children had been exposed to IPV. The most common types of child exposure to IPV were "prenatal," "heard," "seen," and 10.0% of children were physically or verbally involved in IPV. Mothers reported high scores of Total Difficulties on the SDQ in 71.7% of all children exposed to IPV, and teachers in 59.8%. Multivariate logistic regression analysis showed that the strongest association with behavioral difficulties was exposure to IPV in the 1-2 year age range.  IVP - intimate partner violence (VPI- Violência por Parceiro Íntimo)  SDQ - Strengths and Difficulties Questionnaire (SDQ- Questionário de Pontos Fortes e Dificuldades ) |
| Correia et al.  **(2019)[**77] | Monthly income in Brazilian reais and participation status in the Family Allowance Program. Social class was determined by the Brazilian Criteria "Brazil Criteria" and parents' education. | We found that the prevalence of delay for all domains was higher for children aged 36-72 months compared to those under 36 months. Lower socioeconomic status, assessed by monthly income and social class, was strongly associated with an increased risk of developmental delay. There was some indication of lower delay prevalence in poor families that participated in CCT programs compared to those who were eligible but did not participate. |
| Delgado et al.  **(2020)[**78] | Race/color, income, socioeconomic benefit, basic sanitation, local violence, smoking, attendance at daycare, father's presence. | Out of a total of 110 evaluated children, motor performance fell below expected in more than half of them (63.6%, n = 70). There was a statistically significant association between motor development and delayed vaccines (p = 0.005), living with smokers at home (p = 0.047), and receiving socioeconomic benefits (p = 0.036). |
| Rocha et al.  **(2020)[**79] | Age and maternal education, marital status, family income, occupational status, income transfer benefit. | Children exposed to adverse experiences in childhood showed low scores in all five domains of child development measured: communication, gross motor coordination, fine motor coordination, personal and social problem-solving. A higher number of adverse experiences in childhood was linearly associated with lower development scores. Maternal mental health and intimate partner violence were also associated with lower development scores. |
| Souza et al.  **(2021)[**80] | Families were designated as low SES and medium SES based on the preschool they attended: philanthropic daycare or two preschools funded by the municipality that serve families with low socioeconomic status | The most important and intriguing finding of the current work, however, is that, despite lower ToM and vocabulary scores, Brazilian children with low SES actually performed better on the selective trust task than middle-class children attending private schools. |
| Morais et al  (**2021)[**81] | The Brazilian Economic Classification Criteria were used to classify the economic situation of the children's families based on the family's possessions at home and the level of education of the head of the family. (1) belonging to the most deprived economic classes (D and E); (2) low maternal education (not completing high school); (3) single-parent families (only the mother; with or without the presence of other relatives); (4) absence of the father at home; and (5) having three or more siblings. | Motor development variable, none of the four predictors (socioeconomic index, neighborhood, home, or daycare) had a statistically significant impact. Cognitive development variable, predictors socioeconomic index, neighborhood, home, and daycare explained 25% of the variations. However, only home and daycare had a direct, positive, and statistically significant impact, indicating that the better the home and daycare environment, the better the child's cognitive development. |
| Munhoz et al.  **(2022)[**82] | Socioeconomic conditions: (1) Number of children under seven years old living in the household, (2) Self-declared race/color, according to the classification of the Brazilian Institute of Geography and Statistics (categorized as white, brown, black, and others); (3) Maternal education in completed years (0-4, 5-8, and ≥ 9); (4) Living with husband/partner (yes/no); (5) Working outside the home (no, some days, every day). | Infant development scores (total and in all domains) were about 12% lower in preterm and intrauterine growth-restricted (small for gestational age) children. Lower scores were observed in children of mothers with low education, with symptoms of depression, with two or more children under seven years residing in the household, and who did not report self-perceived support/help during pregnancy. |
| **FIELD OF INTEREST - MULTIPROFESSIONAL (n=9)** | | |
| Ferreira et al.  **(2021**)[68] | Residents in indigenous communities. | No birth weight records were found for 26.7% of the 6,128 sampled children in the researched documents. The North region had the highest number of children with no birth weight data (51%), while the other regions had less than 30% missing data, ranging from 18.3% (Northeast) to 26.7% (South/Southeast). The mean birth weight of the 3,994 analyzed children (65.2% of the total sample) was 3,201g (SD ± 18.6g), regardless of gender, type of delivery, and natality. The prevalence of low birth weight was 7.6% (n = 302). Low birth weight ranged from 7.3% in the Northeast to 7.9% in the Center. |
| Chaves et al.  (2005)[83] | The study does not specify how poverty or social vulnerability was characterized. It only reports that it involves residents from a poor region. | Life course events, such as malnutrition and childhood infections during tooth development, may be associated with enamel defects in socioeconomically disadvantaged communities and may compromise oral health-related quality of life. |
| Oliveira et al.  **(2008)[**84] | Family income, parents' education, number of children, own house, overcrowding (number of people and number of rooms). | There was an association between nutritional, socioeconomic factors, and dental caries. Children with low weight and those with adverse socioeconomic conditions and mothers with less than 8 years of education were more likely to have caries, including severe dental caries. |
| Lima et al.  **(2010)[**85] | The study population belongs to an impoverished urban community called Universitary Park. | The prevalence of new parasitic infections, especially by Giardia spp., decreased significantly with vitamin A intervention, suggesting an immunoregulatory modulation of this nutrient on intestinal parasitic infections. |
| Pedraza et al  **(2016)[**87] | Information about children's sociodemographic conditions (age, gender, residential area, Family Allowance Program benefits) was included. | The most frequent anthropometric deviations were statural deficit (7.0%) and overweight (8.3%). Food insecurity was found in 68.9% of families. Anemia was found in 16.97% of children, and zinc deficiency in 13.28%. Children had a high frequency of health problems in the last 15 days reported by the mother (68.6%), eosinophilia (65.5%), parasitism (82.7%), and polyparasitism (46.4%). |
| Marques et al.  **(2020)[**88] | Residents of two riverside communities formed by fishermen and rubber tappers in the Amazon region. The study cited family characterization: family income, maternal education, number of family members. | The high prevalence of intestinal parasitoses is an important finding, drawing attention to the low environmental conditions and neglect that threaten the future of these Amazonian children. In the social transition occurring in Western Amazon, moderate anemia (Hb < 11.0 g/dL) is high and unevenly distributed between the two communities. Moderate anemia and mild to moderate malnutrition had a higher frequency in children from Itapuã than in children from Bom Futuro. This indicates that there are environmental modifying factors that differentiate these groups. |
| Silveira et al.  **(2021)[**89] | Children in shelters.* The socioeconomic condition was not provided, but the reasons for admission to the institution were classified as: intrafamily violence (physical or psychological), sexual abuse, sexual exploitation, negligence, or abandonment." | Stress had a negative impact on the health of the studied sample. Asthma was the most prevalent disease, which is the main cause of hospitalization among Brazilian children and is positively associated with stress. |
| **FIELD OF INTEREST – DENTISTRY (n = 7)** | | |
| Dini et al.  **(2000)[**91] | Absence of breastfeeding, social class, and mothers' education. | Dental caries were observed in 46% of children; 17% of them had the most extensive pattern involving molars and incisors. Social class, mother's education, and the age at which breastfeeding was discontinued showed statistically significant associations with dental caries. Baby bottles with added sugar were still given to 80% of the children. When significant variables were considered, only the age at which breastfeeding ended showed a significant relationship with the disease pattern. Children who were never breastfed or were breastfed beyond 24 months had a higher prevalence of the most extensive caries pattern. |
| Rodrigues et al. **(2001)[**92] | Income, economic class, education, and occupation of the head of the family. | Children attending nurseries that did not adopt guidelines on reducing sugar intake had a higher risk of caries, with an odds ratio of 3.6 compared to those attending nurseries with guidelines. Children who had more than 32.6 g of sugar daily in the nursery were 2.99 time more likely to have a high caries increment than those with less than that amount. |
| Peres et al.  **(2003**)[93] | Per capita family income; Gini coefficient, and literacy rate among individuals over 20 years old. | A higher dmft index was associated with a low infant development index, a high illiteracy rate, and non-fluoridated water supply. The infant development index was significantly associated with the care index, and the number of dentists in public service per 10,000 inhabitants showed borderline statistical significance.  dmft – dentes decíduos |
| Ferreira et al.  **(2007)[**94] | Level of education and occupation of both parents, number of siblings, and family income. | 40% of children presented dental caries [mean deft (SD): 1.53 (2.75) ]. The deft increased with age (P < 0.001) and was significantly higher in children of mothers with low educational levels and low family income. The greatest increase in the prevalence and severity of caries occurred between the ages of 1 and 2 years. Logistic regression showed higher odds of dental caries with mothers with less than 4 years of education.  SD - mean deft * |
| Lima et al.  **(2016)[**95] | Income, public and private school. | 42% of high SES children and 2.7% of low SES children used fluoride-free toothpaste or toothpaste with a concentration below 1,000 ppm F. Fluoride intake was associated with SES, brushing frequency, amount of toothpaste used, and fluoride concentration in toothpaste (P < 0.05). Low SES children had higher fluoride intake during brushing (0.045 mg F/kg body weight/day), compared to high SES children (0.023 mg F/kg body weight/day). Unlike low SES children (68.9 percent), all high SES children were free of caries. |
| Cangussu et al. **(2016)[**96] | Number of children under seven years old residing in the household, Race; Maternal education in completed years; Living with husband/partner; Working outside the home, daycare (public/private/philanthropic); Education and occupation of the mother and father; Mother and father's occupation (low qualification/unemployed); Per capita household income; Number of siblings; Number of people per room; Running water in the residence; Housing condition. | The mean decayed teeth was 0.18 (SD = 0.75) at the beginning of the study and 0.55 (SD = 1.40) at the end. The incidence in caries-free children was 18.5%, and in the total group, it was 22.6%. Social vulnerability was a strong risk factor for caries in children under two years. |
| Antunes et al.  **(2020)[**97] | Clinical conditions; CCEB (Brazilian Economic Classification Criteria), maternal education, daycare location (urban or rural).  CCEB - Brazilian Economic Classification Criterion (Critério de Classificação Econômica Brasileiro CCEB) | The prevalence of Traumatic Dental Injuries (TDI) was 17%. Children aged 37 to 60 months (P = 0.04; OR = 0.59 CI 95% 0.34-1.01) were associated with TDI. The average impact on Oral Health-Related Quality of Life (OHRQoL) in the group with or without TDI was low without statistical difference (P = 0.97).  TDI – Traumatic Dental Injuries (Traumatismo Dentário) |
